# Beyond the Petri Dish: Exhibitions as Catalysts for Microbial Literacy—Bridging Science, Culture and Society

**DOI:** 10.1111/1751-7915.70222

**Published:** 2025-08-20

**Authors:** Rachel Armstrong

**Affiliations:** ^1^ KU Leuven Leuven Belgium

## Abstract

Microbes orchestrate Earth's biosphere, yet public understanding of their essential role in sustainability, health and social equity remains limited. Traditional microbiology education often fails to engage diverse audiences, perpetuating gaps in societal decision‐making. This opinion piece argues for expanded microbial literacy through interdisciplinary, experiential learning, with exhibitions proposed as critical platforms to bridge science, culture and society. Drawing on the International Microbiology Literacy Initiative (IMiLI) mission, the contributions of spatial and narrative‐driven encounters are considered—from ancient memory palaces to modern theatres of microbial activity—in transforming otherwise abstract microbial processes into tangible, transferrable, actionable knowledge. *Individual case studies of historic and contemporary exhibitions such as We the Bacteria: Notes Toward Biotic Architecture* are examined through a curatorial vision to understand how the relationship between people and microbes can be shaped through experiential knowledge while advancing microbial literacy. However, such initiatives require careful balancing of innovation with ethical communication to avoid reductive or misleading narratives. Scaling these approaches through global collaboration between scientists, educators and designers—aligned with IMiLI's vision of lifelong, learner‐centric microbiology education—could effectively engage audiences who have limited access to scientific knowledge, resources, or engagement opportunities and support progress toward UN Sustainable Development Goals.

## Graphical abstract

Microbes shape our health and environment, yet remain invisible to most people. Exhibitions use art, design and storytelling to make microbial science tangible and engaging. By turning abstract processes into sensory experiences, they promote sustainability, health equity and global collaboration—aligning with lifelong learning and the UN Sustainable Development Goals.

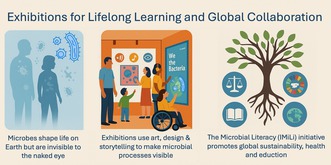

## 1 Introduction: Expanding Microbial Literacy Through Complementary Knowledge Systems

This article explores how microbiology education can evolve beyond traditional scientific communication by adopting a curatorial vision—showcasing how exhibitions can bridge science, culture and society. Public misunderstandings about microbes' role in health, climate and inequality pose significant societal risks, as misinformation continues to spread (Timmis et al. 2024). While empirical science remains foundational—driving scientific, technological and medical progress (Timmis et al. 2024)—knowledge is also shaped by experience, values and context. These dimensions, often overlooked in conventional science communication, are essential for meaningful public engagement (Polanyi 1966; Timmis et al. 2019). *Complementary epistemologies—such as spatial reasoning, embodied interaction and narrative‐based learning—can enrich microbiology education. These approaches* align with the goals of the International Microbiology Literacy Initiative (IMiLI), which promotes global microbiology education through accessible, student‐centred resources designed to cultivate critical and systems thinking (Timmis et al. 2019, 2024). Although distinct from empirical methods, *design and artistic practices can enhance microbial literacy by engaging the senses and emotions*, offering powerful ways to connect science with diverse audiences (Timmis et al. 2024). Drawing from philosophy, history and design, this article examines how exhibitions and performative strategies can bridge the gap between laboratory science and public understanding through lived experience. Understanding the nature of knowledge—a central concern of epistemology—can deepen insight and enable innovation beyond raw data (Nelson 2020).

## 2 Exhibitions as Tools for Experience‐Based Microbiology Learning

**FIGURE 1:**
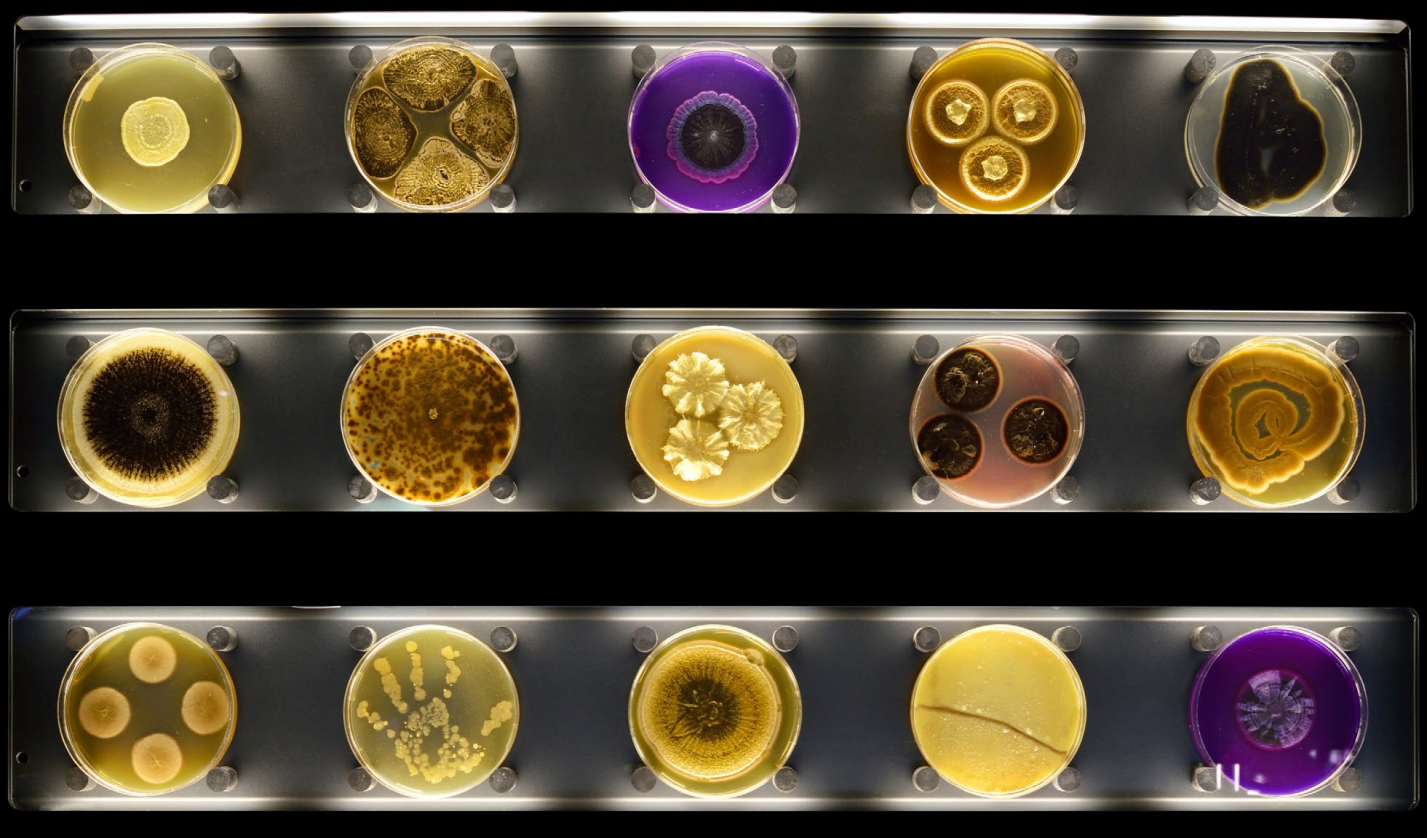
Three rows of microbial Petri dishes on display at ARTIS‐Micropia in Amsterdam, courtesy of The Netherlands Museums Association, 2025.

Exhibitions can serve as powerful tools for translating complex scientific ideas into accessible, engaging formats. Historically, they have bridged specialised knowledge and public understanding—from Renaissance memory theatres to 19th‐century hygiene displays (Timmis et al. 2019). Today, exhibitions use spatial, sensory and performative techniques to make microbial topics—such as nutrient cycling or atmospheric change—more relatable. Permanent exhibitions like ARTIS‐Micropia (Amsterdam, 2014)—the world's first museum dedicated to microbes—use interactive displays, microscopes, live cultures and gamified labs to promote microbial literacy by combining scientific content with participatory engagement in ways that resonate with everyday human experiences (Timmis et al. 2024) (Figure 1).

To broaden their reach, collaborations between museums and educational institutions can leverage scalable tools—such as virtual tours or low‐cost, hands‐on resources like foldable microscopes embedded with QR codes for guided exploration. However, for these initiatives to be truly impactful, barriers related to cost, digital access and educational equity must be addressed. When implemented thoughtfully, such approaches not only enhance public understanding of microbiology but also contribute to addressing systemic challenges like educational inequality—highlighting the transformative potential of inclusive science communication (Timmis et al. 2024).

## 3 Types of Learning and the Challenge of the Invisible

The challenge of microbial literacy lies in the paradox of microbes: though they govern Earth's biosphere, their imperceptibility has historically made their existence a matter of belief rather than direct experience. Before microscopes, concepts like *miasma* or the ‘invisible hand’ of disease reflected attempts to explain unseen phenomena. Even after Antonie van Leeuwenhoek's 17th‐century discovery of ‘animalcules,’ public scepticism persisted, as these findings competed with folklore and pseudoscience. This tension highlights a dilemma: scientific proof alone cannot ensure understanding when the subject remains beyond sensory perception. The invisibility of microbes, combined with their deep integration into complex environments, limits what we can learn through direct observation alone. Their behaviours change with context, their metabolic functions often defy visual cues, and their collective actions emerge only at larger scales. Understanding microbial life, therefore, requires learning strategies that go beyond empirical instruction, such as by incorporating systems thinking, modelling, sensory engagement and conceptual frameworks that reveal hidden patterns and relationships. Microbial education must integrate other knowledge systems that offer *spatial* and *experiential* learning; approaches that are shaped by context and direct interaction, not just information.

### 3.1 Spatial Mnemonics and the Memory Palace Tradition

**FIGURE 2:**
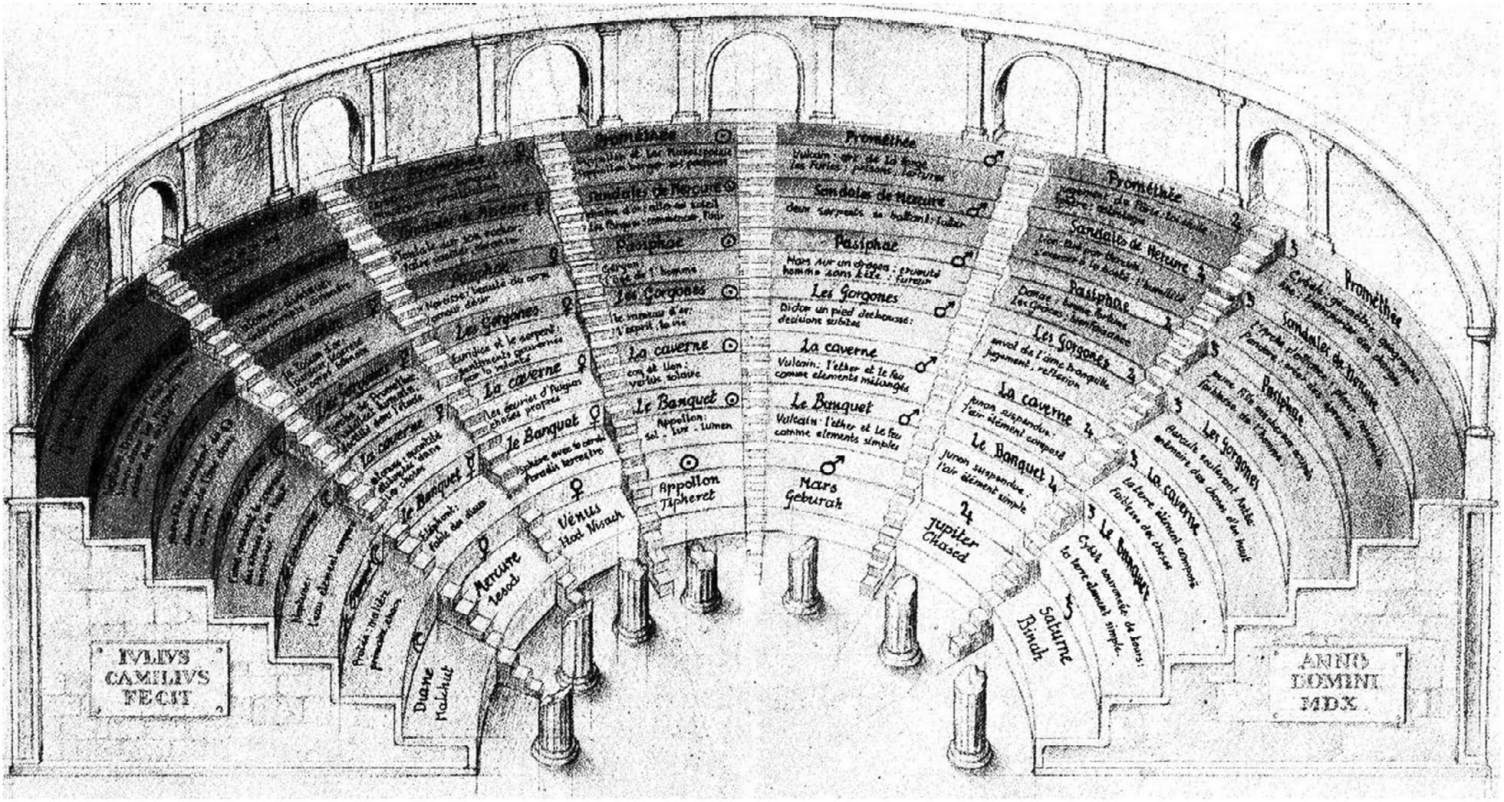
The Memory Theatre of Giulio Camillo (1550).

The ancient method of loci, attributed to Simonides of Ceos (556–468 bce), offers a foundational model for spatial learning. It links information to locations in a mental ‘map’—like placing memories along a familiar path—for easier recall. According to legend, Simonides identified victims of a collapsed banquet hall by recalling their seating, showing that memory is spatial (Haller 2014). This insight was later formalised in Renaissance ‘memory theatres,’ where Giulio Camillo's *Theatro della Memoria* (1544) arranged classical knowledge in architectural form. Users could mentally ‘walk through’ symbolic tiers, from mythological figures to planetary spheres (Figure 2) (Yates 1966). *Camillo believed that all human concepts could be retained and mastered* through *loci and images using spatial navigation* and embodied *encounter*.

### 3.2 Gibson's Affordances: The Ecology of Perception

James J. Gibson's theory of affordances (1979) suggests that organisms perceive their surroundings in terms of possible actions (‘affordances’) rather than abstract information. For example, a staircase is seen as ‘climbable’ based on what the body can do, not from a prior analysis. This idea has important implications for how microbial exhibitions are presented. If perception is relational, then promoting microbial understanding requires creating spaces where microbes' roles encourage interaction. These affordances can be designed for both microbes (like in bioreactors) and for audiences—through hands‐on experiences, physical models, storytelling and sensory elements like textures or sounds. These strategies help connect human perception with microbial worlds, turning abstract theory into something people can feel and understand.

## 4 Exhibitions as Experiential Classrooms

**FIGURE 3:**
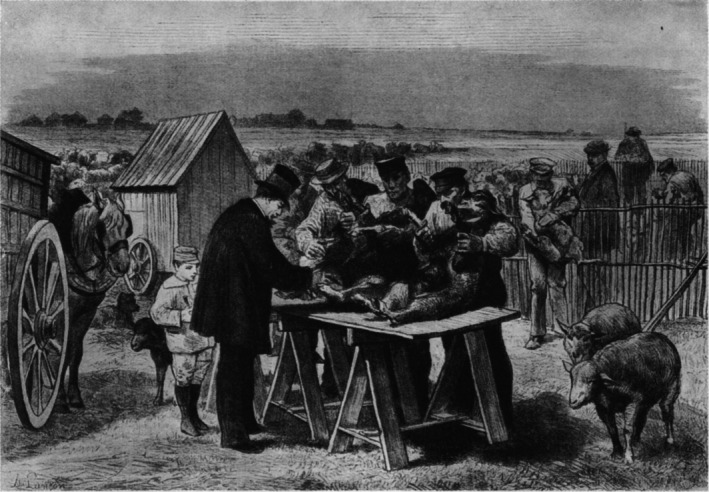
Louis Pasteur vaccinating sheep in Pauly‐le‐Fort on 31 May 1881 (De Kruif 1927).

While the arts and humanities excel at engaging unconventional knowledge, science has also effectively adopted these approaches. A notable example is Louis Pasteur, who advanced germ theory and used performative strategies to communicate his findings. In 1881, Pasteur staged a dramatic anthrax vaccine trial at Pouilly‐le‐Fort before journalists, sceptics and scientists (Figure 3). He vaccinated twenty‐five sheep, left another group untreated and exposed both to anthrax. The vaccinated sheep survive; the others died—visibly validating his hypothesis. This event made microbiology visible and persuasive by turning scientific claims into public performance (Latour 1988).

**FIGURE 4:**
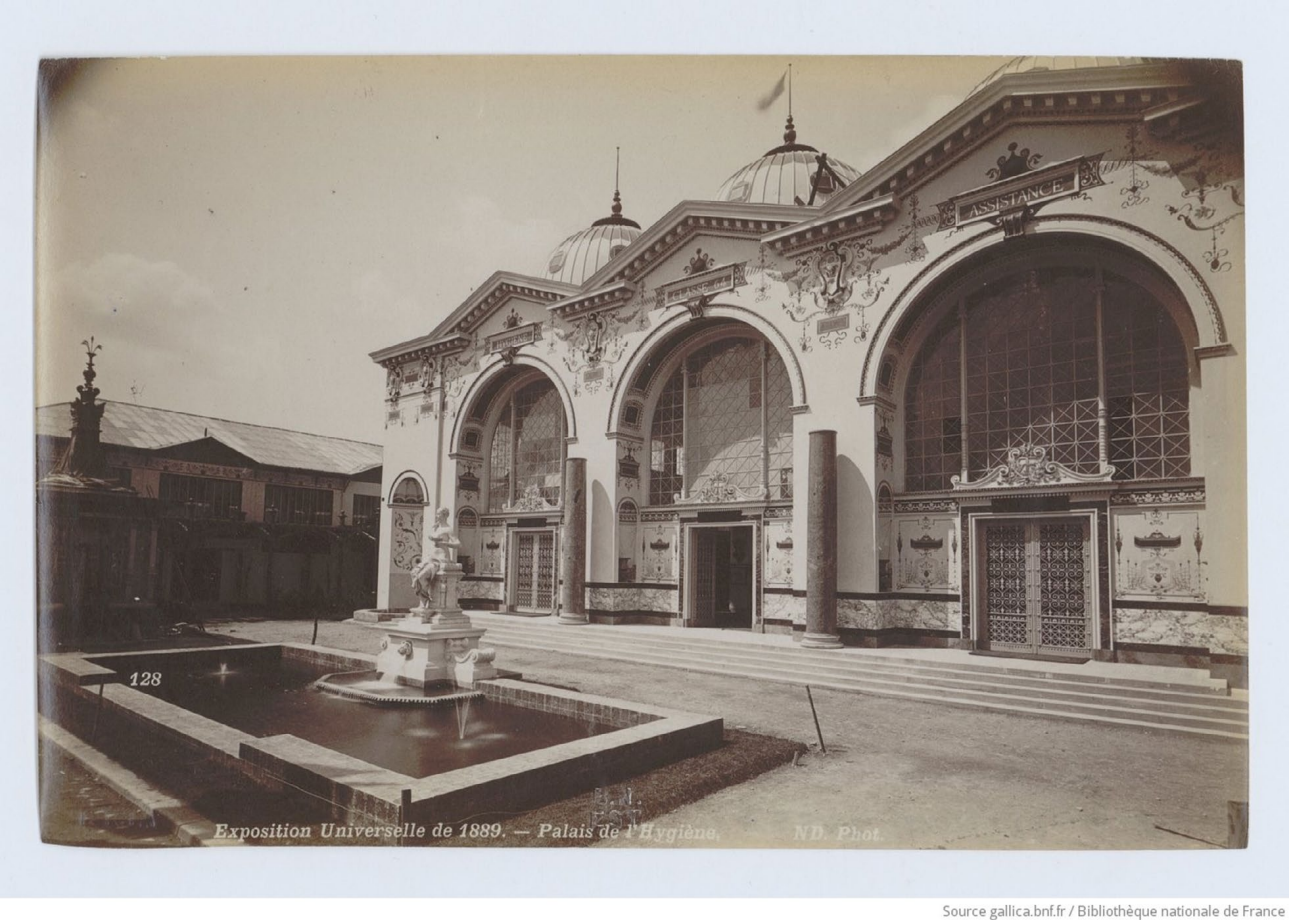
Palace of Hygiene, Exposition Universelle de 1889 [Photograph]. Bibliothèque nationale de France, Gallica (2025).

Pasteur's use of unconventional methods helped establish his legacy—even influencing architecture. While the Eiffel Tower showcased industrial progress at the 1889 Paris Exposition Universelle, the nearby Palais de l'Hygiène, styled like Roman baths (Figure 4), linked modern hygiene to civic ideals (Librairie Illustrée 1889). The palace promoted germ theory by turning microbes into a public message (La Berge 1992). Exhibits featured sanitation technologies—like porcelain toilets, hydrotherapy tools and sewer system models labelled ‘Tout‐à‐l'égout’ (Everything to the sewer)—highlighting Paris' infrastructure reforms. A particularly illustrative moment involved the real‐time clarification of turbid water using the Chamberland filter, a device developed as part of Louis Pasteur's foundational contributions to microbiological science. These interactive displays made the invisible microbial world tangible and reframed hygiene as a shared civic duty grounded in microbiology (La Berge 1992).

### 4.1 From Germ Warfare to Microbial Kinship: Reimagining Our Invisible Relationships

Science communication often simplifies complex ideas, sometimes at the expense of nuance. Although Sergei Winogradsky highlighted microbes' ecological roles in the 19th century (Dworkin and Gutnick 2012), public narratives cast them as enemies. This ‘war on germs’ shaped 20th century culture—from soap advertisements to hospital design—favouring sterilisation over understanding. By mid‐20th century, this mindset extended to antibiotics, pesticides and disinfectants. Rachel Carson's *Silent Spring* (2002) exposed the ecological costs, raising environmental awareness, though microbes remained misunderstood. Contemporary microbiome research increasingly reveals most microbes as essential allies—symbionts, recyclers and architects of life. The human gut microbiome, for instance, plays a critical role in regulating immunity and metabolism (Valencia et al. 2025). Similarly, skin‐associated microbiota contribute to barrier integrity, immune homeostasis, pathogen defence and wound healing (Harris‐Tryon and Grice 2022). Beyond the human body, environmental microbiomes are equally vital: they sustain soil fertility (Timofeeva et al. 2023) and influence the composition of the air we breathe (Górny 2020). Collectively, these microbial communities are indispensable partners in maintaining planetary health (Margulis 1998).

## 5 The Microbial Theatre: From Metaphor to Material Encounter

A new kind of microbial theatre is emerging—one that is not just metaphorical. In 1988, John Whipps and colleagues described microbial ecosystems as a ‘theatre of activity’ (Whipps et al. 1988), emphasising their dynamic, context‐driven nature. Microbes are not isolated actors but part of ecological scripts, shaping and responding to their environments. This theatrical lens aligns with traditional theatre as a space of encounter. Visitors engage through touch, small and taste—handling fermented materials, sensing cultures, or preparing food (Griffiths 2013). These embodied experiences turn exhibitions into ‘memory theatres,’ where knowledge is felt as well as learned. Such performative strategies challenge outdated views of microbes as mere pathogens, instead presenting them as symbionts and ecological agents. The theatrical metaphor becomes a literal curatorial tool, shaped by curatorially deigned affordances to promote microbial literacy using sensory‐rich, scientifically grounded encounters with microbes. The following section examines how curators are drawing on unconventional forms of knowledge to confront these challenges—making microbial life perceptible and transforming exhibitions into spaces where microbial agency, deep time and environmental entanglement become intelligible.

### 5.1 Engaging the Imperceptible

How do exhibitions render the invisible world of microbes perceptible? Beyond explanation, they employ immersive simulations and digital technologies to transform microbial processes into vivid, multisensory experiences through (i) physical simulation and immersion; (ii) insights using digital media; (iii) environmental embeddedness; (iv) microbial versus human timescales, which are discussed in more detail in the following sections.

#### 5.1.1 Physical Simulation and Immersion

**FIGURE 5:**
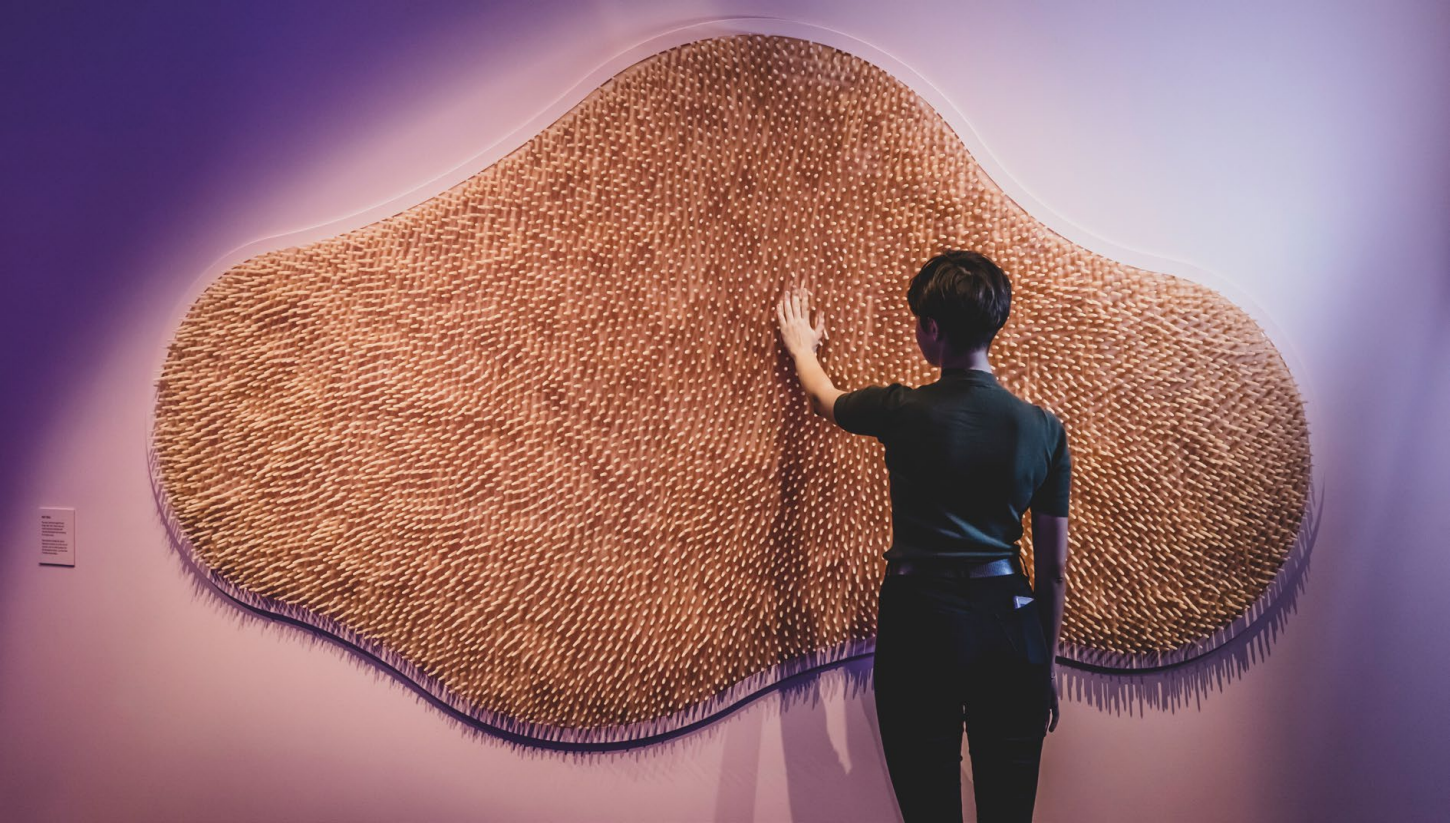
Welcome to Your Gut wall panel in the Gut Feeling exhibition in Melbourne Museum, photograph Rob Zugaro courtesy of the Melbourne Museum press pack, 2020.

The *Gut Feelings: Your Mind, Your Microbes* exhibition (Museums Victoria 2020) combined microbial science with sensory design (Griffiths 2013). In partnership with the University of Melbourne and the Peter Doherty Institute, over 1500 visitors contributed saliva and lifestyle data, generating a live microbiome dataset (Time Out 2020). Visitors walked through a ‘gut tunnel,’ where light, sound and texture simulated digestion. Gamified stations let them ‘feed’ their microbiome, while textured walls mimicked intestinal villi (Figure 5) (Museums Victoria 2020). Live projections of microbiome data created visceral links between body and microbe; the *State of Our Microbes* display translated research into relatable insights—showing, for example, that high‐carbohydrate diets were linked to 23% lower microbial diversity, and 88% of adults ate too few vegetables (Wali et al. 2021). Since the data came from the visitors themselves, the exhibition became a dialogue—revealing how everyday choices shape our inner ecosystems.

*Gut Feelings* launched at a paradoxical moment—just after COVID‐19 lockdowns, amid strict hygiene protocols. While the exhibition imagined microbes as allies, public health measures emphasised their threat, highlighting tensions between microbial intimacy and sanitation. The show pioneered a citizen science model, inviting visitors to contribute microbiome data. Yet, participation gaps revealed how socioeconomic factors shape access to science. Inclusivity, it showed, must be designed—not assumed.

Despite these challenges, *Gut Feelings* advanced public understanding of microbes as multifaceted entities—simultaneously symbionts, pathogens and environmental agents—aligning with broader initiatives like IMiLI to promote microbial literacy in support of health, sustainability and informed decision‐making.

#### 5.1.2 Insights Using Digital Media

**FIGURE 6:**
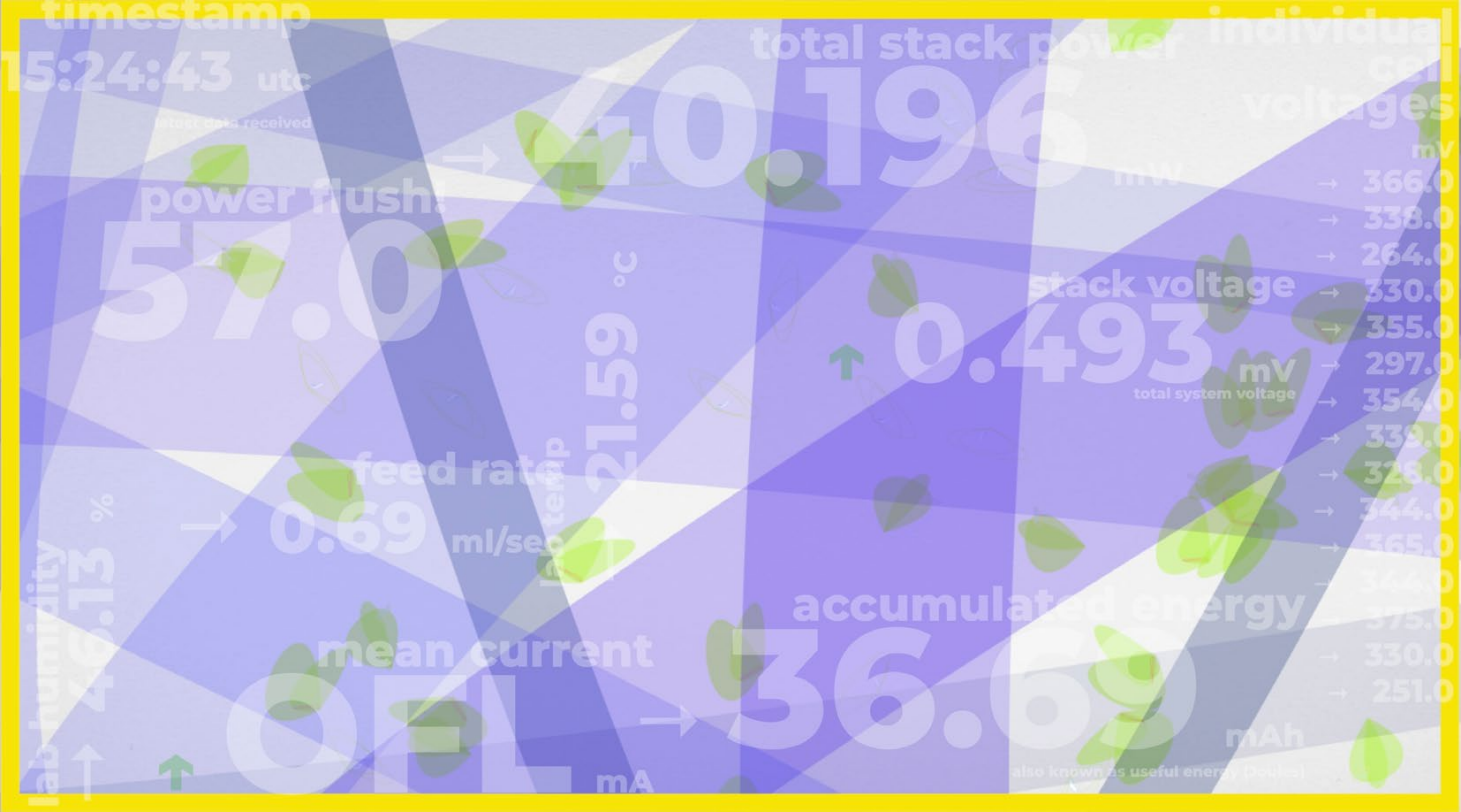
Anaerobic biofilm activity in microbial fuel cells (MFCs) is visualised through sound and animation. Visitors interact by feeding or warming the biofilm via screen buttons. Artwork by Julie Freeman, courtesy of the ALICE consortium (Ioannis Ieropoulos, Rachel Armstrong, Julie Freeman).

Digital platforms are transforming microbial exhibitions by making microbes visible through their metabolisms. The *Active Living Infrastructure: Controlled Environment* (ALICE) prototype promotes interspecies connection—like caring for a pet or houseplant—through real‐time biodata sonification and generative visuals (Figure 6) (ALICE Interface 2021).

**FIGURE 7:**
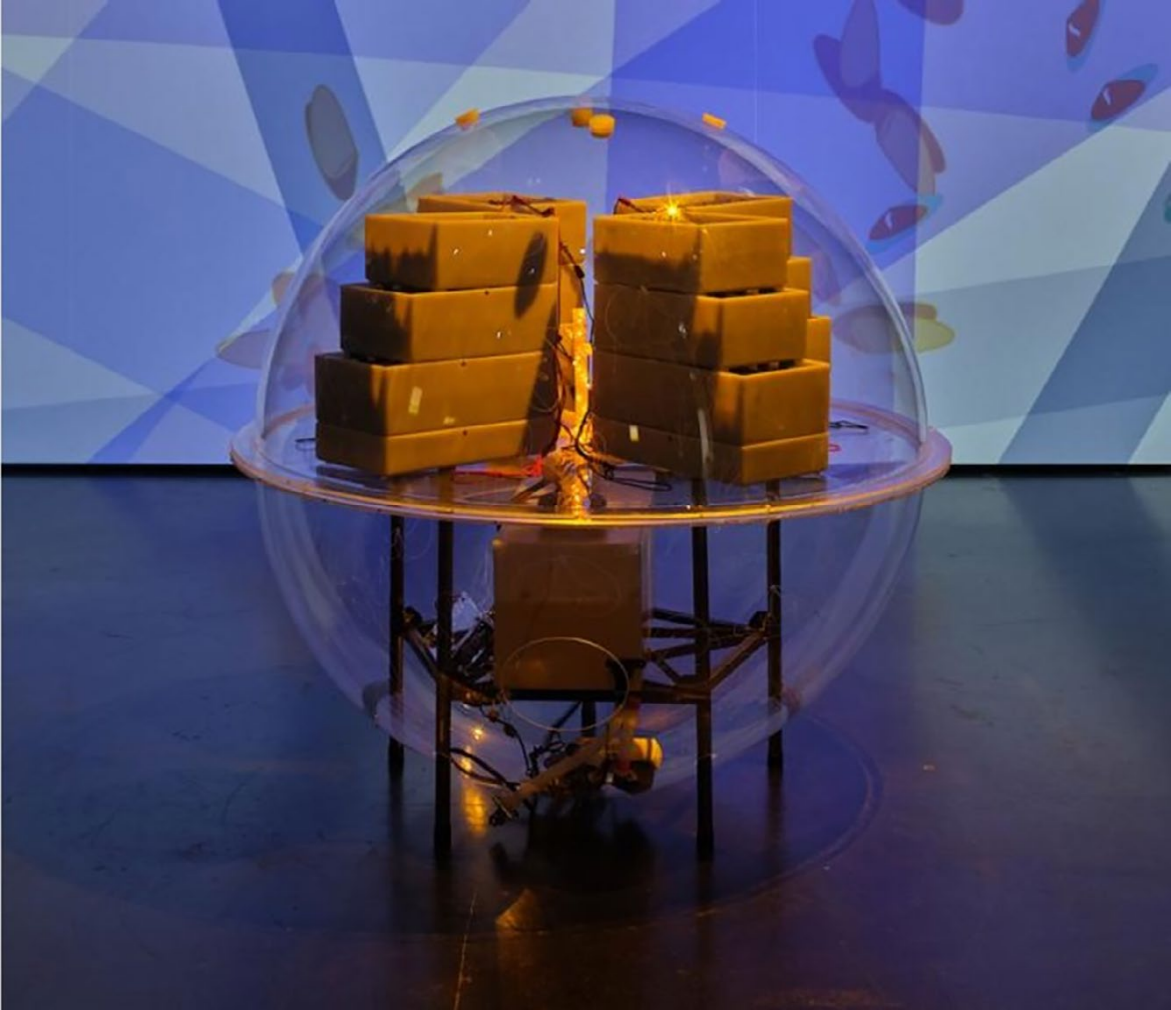
ALICE installation with 15 MFCs at ZKM gallery, Karlsruhe. Data visualisations by Julie Freeman, courtesy of the ALICE consortium.

ALICE incorporates microbial fuel cells (MFCs) into its infrastructure, where organic matter is metabolised by electrogenic microbes to generate electricity (You et al. 2022). These outputs are translated into soundscapes and animations, making microbial interaction sensorially engaging (Figure 7). A digital dashboard displays metrics like energy yield and CO_2_ reduction, linking user actions to environmental outcomes. This feedback loop positions microbes as active environmental agents, with humans as co‐participants in shared microbial ecologies. Like *Gut Feelings*, ALICE combines embodied interaction with empirical data, advancing microbial literacy by connecting microbiology to sustainable living (Timmis et al. 2024).

By staging microbial processes as participatory performances, ALICE transforms the microbial ‘theatre of activity’ into a tangible learning experience. Visitors engage directly with electroactive biofilms—modulating conditions such as nutrient availability and gentle warming—to observe microbial responses in real time. Through digitally mediated feedback, this interaction becomes a kind of software‐facilitated dialogue, allowing participants to explore microbially occupied environments and develop a deeper emotional and cognitive connection with microbial life.

#### 5.1.3 Environmental Embeddedness

Microbial exhibitions face a core challenge: their subjects operate at scales, speeds beyond human perception. To bridge this gap, effective exhibitions embed microbial stories within familiar ecological settings—soil, water and the human body—making them relatable and relevant. This approach dissolves the boundary between observer and microbe, revealing a shared ecological network and multiple dependencies essential to microbial literacy.

**FIGURE 8:**
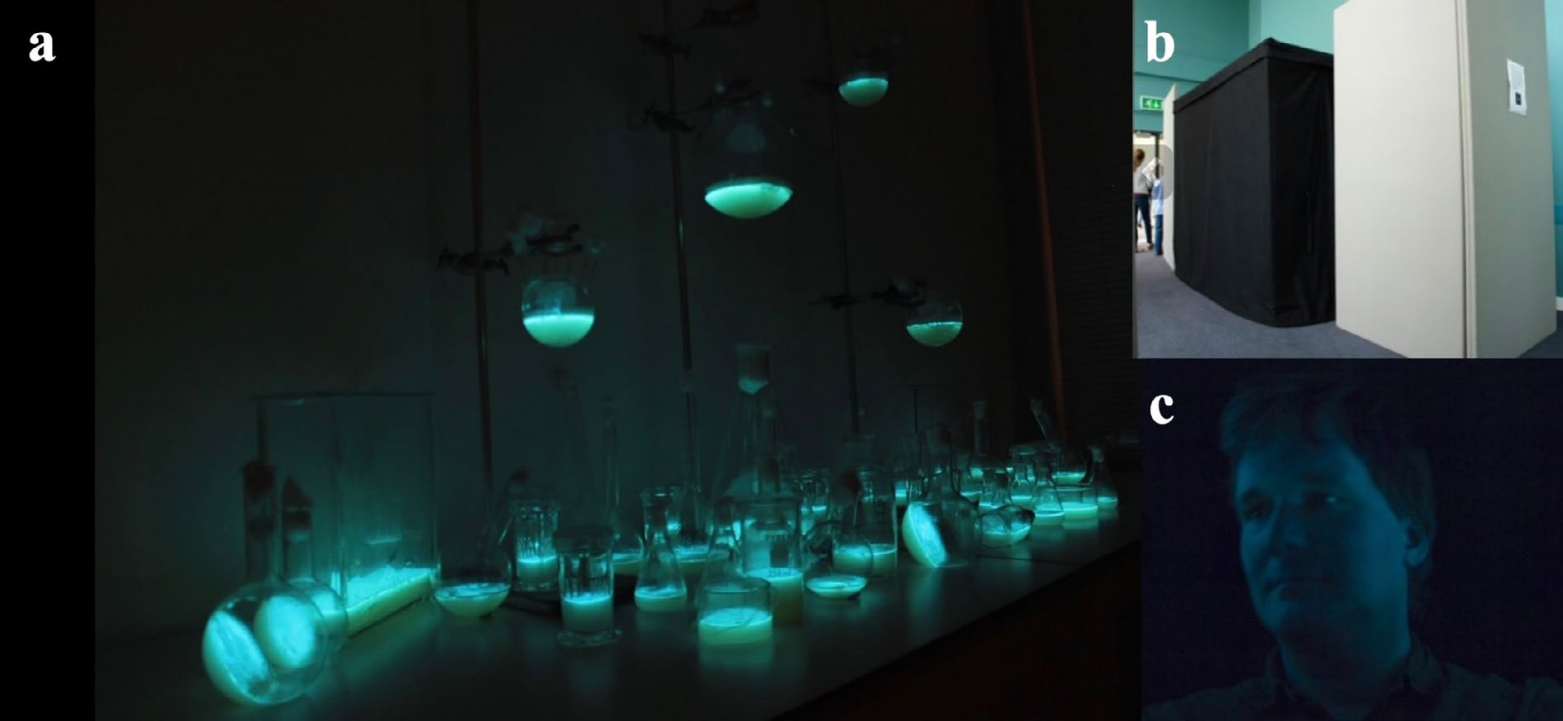
Bioluminescent Photobooth development: (a) preparation of *Photobacterium phosphoreum* ; (b) booth construction at Surrey University (2009); (c) self‐portrait by Simon Park using bacterial light. Courtesy of the artist (Park 2025).

The *Exploring the Invisible* project (2008–2011), a collaboration between artist Anne Brodie, microbiologist Simon Park and curator Caterina Albano, used bioluminescent *Photobacterium phosphoreum* to explore microbial life through light (Figure 8) (Brodie, n.d.; Brodie 2025).

Participants entered a darkened Bioluminescent Photobooth, where their portraits were taken using only the glow of living bacteria. This fading blue‐green light revealed the human form through microbial vitality, prompting calm and reflection. Informally observed and recorded participant reactions revealed a change in perception—microbes were no longer viewed solely as contaminants, but increasingly as cohabitants. By staging these interspecies encounters in venues such as the Old Operating Theatre in London, the project expanded the potential for microbial literacy through memory, empathy and affect. It also demonstrated how interdisciplinary collaboration can render microbial life visible, emotionally resonant and enduringly memorable (Myers 2012).

#### 5.1.4 Microbial Versus Human Timescales

**FIGURE 9:**
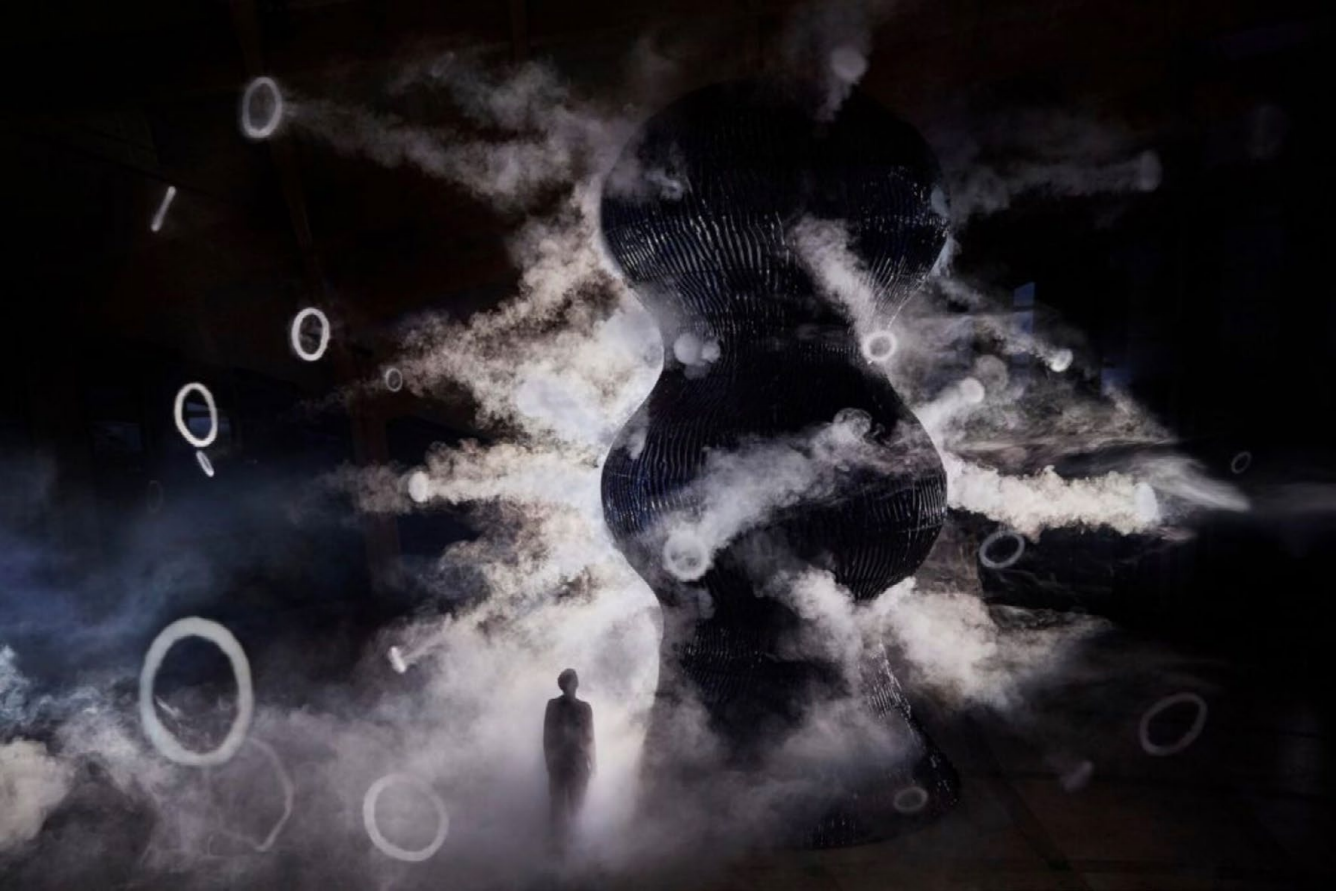
Infinity Blue, Studio Swine, Eden Project. Courtesy of the artists 2018.

Microbial life unfolds across timescales far beyond human perception. To make this comprehensible, exhibitions embed microbial time within familiar milestones—like the rise of oxygen or the evolution of multicellular life—revealing microbes as foundational to life on Earth and essential to microbial literacy. The *Invisible Worlds* exhibition at the Eden Project illustrates this through ∞ *Blue* (*Infinity Blue*), an 8.5‐m ceramic sculpture by Studio Swine that pays tribute to cyanobacteria—oxygen‐producing microbes that transformed Earth's atmosphere during the Archean and Proterozoic eons through the Great Oxidation Event (Figure 9) (Studio Swine 2018; Eden Project 2025). Towering over visitors, the sculpture challenges our bias toward large, complex organisms (Hird 2009), repositioning microbes as central to planetary history. Made from Cornish clay, it emits scented vapour rings—developed with Givaudan perfumers—to evoke Earth's evolving atmospheres.

Through scent, scale and sculptural form, ∞ *Blue* translates evolutionary time into a multisensory aesthetic experience—reminding audiences that the air we breathe, the ecosystems we inhabit and the bodies we live in are shaped by microbial legacies. The work demonstrates how aesthetic strategies can evoke microbial symbiosis and deep time in ways that are both emotionally resonant and scientifically grounded.

### 5.2 Rewilding Microbes: The 3D Probiotic Petri Dish

Microbial exhibitions are transitioning from containment to cohabitation. Where microbes were once sealed behind glass, new curatorial approaches now invite participation—transforming galleries into living laboratories that reflect our shared microbial environments. This transition is exemplified in *We the Bacteria: Notes Toward Biotic Architecture* at the Milan Triennale, curated by Mark Wigley and Beatriz Colomina (Milano 2025). Here, the gallery becomes a three‐dimensional, probiotic Petri dish, immersing visitors in microbial atmospheres and environments rather than isolating them.

**FIGURE 10:**
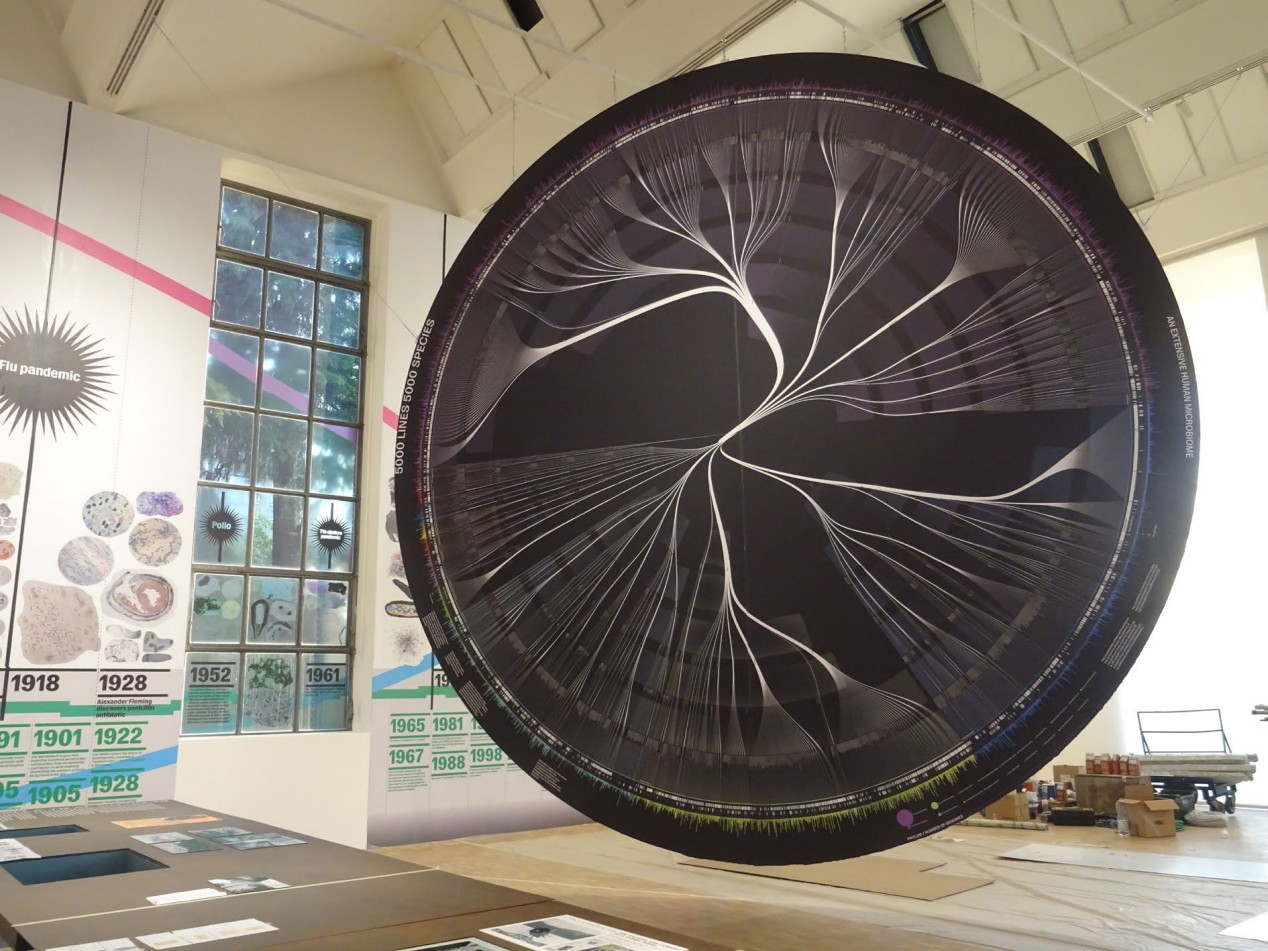
Two Sides of the Same Coin, human microbiome graphic by Laura Kurgan, Dan Miller and Adam Vosburgh, photograph by Rachel Armstrong, Milan Triennale, 2025.

The visitor's journey begins with the familiar—everyday materials and rituals such as cleaning, eating and breathing. These sensory‐rich environments gradually reveal that microbes are not only all around us but also within us: the *human microbiome* plays a vital role in regulating immunity, metabolism and mental health (Davis 2016; De Luca and Shoenfeld 2019; Radjabzadeh et al. 2022), while environmental microbes influence everything from soil fertility to air quality. A key exhibit, *Two Sides of the Same Coin* by Laura Kurgan, Dan Miller and Adam Vosburgh, traces microbial life from Earth's origins to speculative futures in space (Figure 10). This timeline reframes architecture through a microbial lens, inviting reflection on our evolutionary entanglement with microbial life and the possibilities of engineered symbiosis.

As visitors encounter juxtapositions of objects, images and microbial data, they begin to see how microbes are implicated in a host of everyday occurrences—from the deterioration of historic architecture to the innovation of new materials like bioplastics. This unfolding awareness challenges anthropocentric views of health, design and the environment—reframing the built world as an active participant in microbial ecologies. In this context, bodily and environmental health emerge as multispecies concerns, and microbes are repositioned as co‐creators of our living spaces, rather than passive inhabitants.

This conceptual shift culminates in nine commissioned installations, each exploring a different strand of research into the role of microbes in the built environment and their potential to reshape how we live. Positioning microbes as co‐designers of future habitats, the individual works are explored in the following sections through three thematic lenses: (i) soil as archive, (ii) architecture as microbial partner and (iii) multispecies cohabitation. Together, they transform the gallery into a memory theatre of microbial intelligence—inviting direct, affective and intellectual encounters with the microbial world.

#### 5.2.1 Soil as a Living Archive

These installations treat soil and sewage as microbial archives—revealing how microbes reflect histories of care, neglect and ecological entanglement.

##### 5.2.1.1 Paolo Tavares With Studio Autonoma—*Terra Preta/Dark Earth*

**FIGURE 11:**
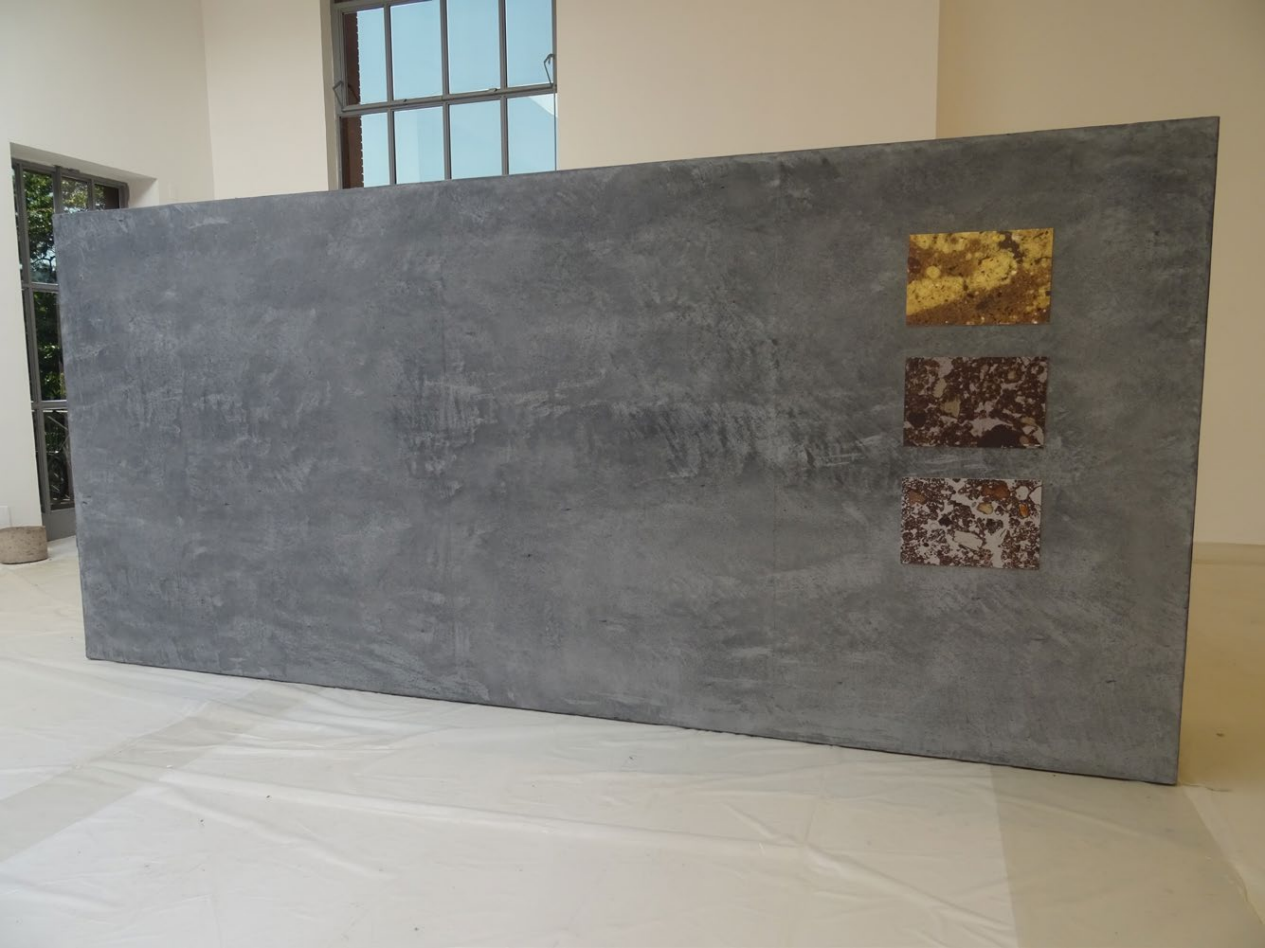
Terra Preta by Paolo Tavares and Studio Autonoma: Rammed earth walls with biochar echo Amazonian Dark Earths, critiquing industrial agriculture while showcasing Indigenous soil stewardship. Photograph by Rachel Armstrong, Milan Triennale, 2025.

This installation uses rammed earth walls embedded with biochar to echo Amazonian Dark Earths (Figure 11). *Terra Preta* critiques the commodification of Indigenous soil knowledge, while highlighting its microbial richness and climate repair potential. It contrasts Indigenous agroforestry with the depleted soils left by industrial agriculture.

##### 5.2.1.2 Philippe Rahm—*Climate‐Shifted Playgrounds*

**FIGURE 12:**
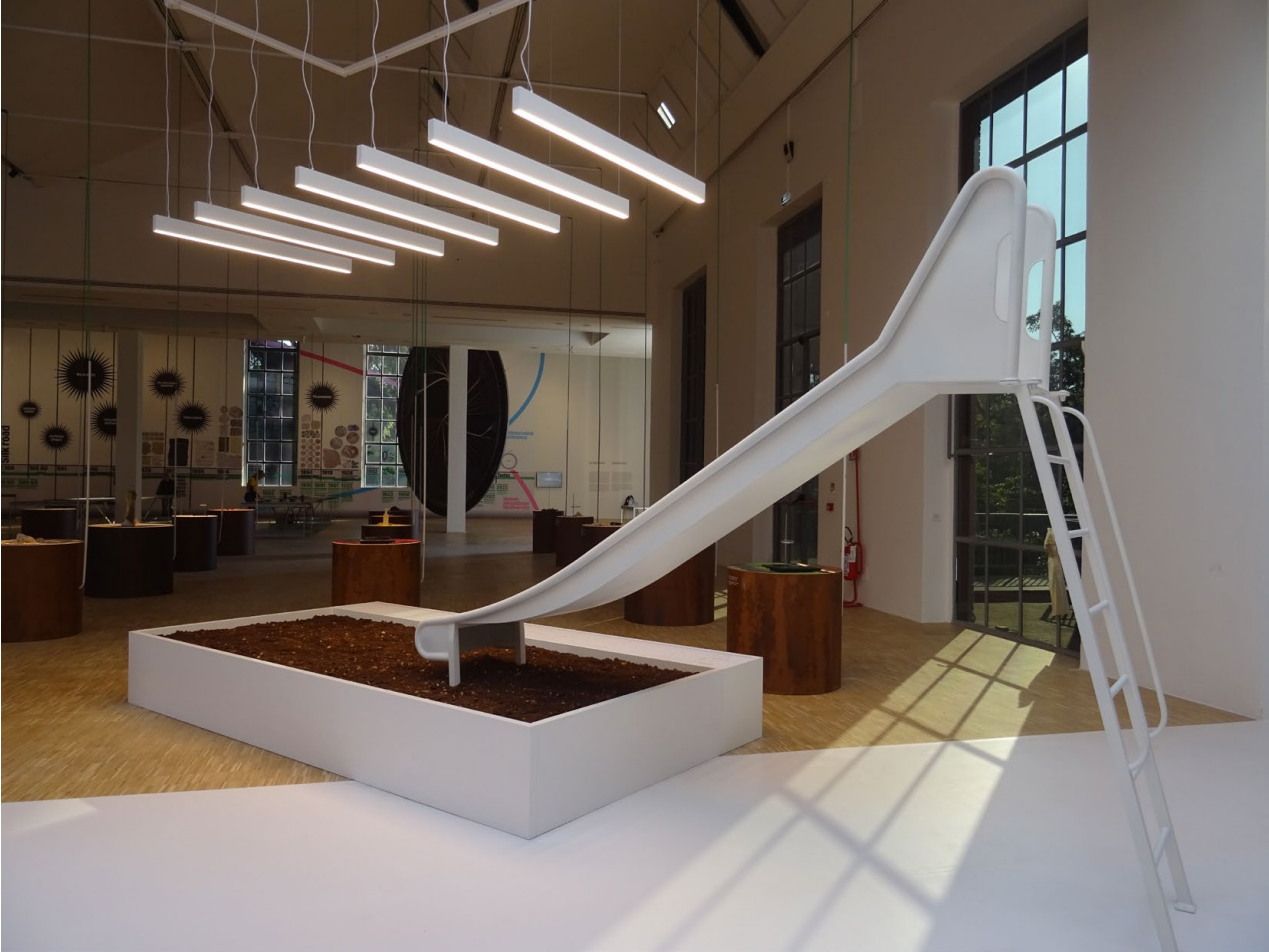
Climate‐Shifted Playgrounds by Philippe Rahm: Transplanted soils from Puglia create ‘pre‐adapted’ play spaces, using microbial communities from warmer climates to prepare for Milan's projected +5°C future. Photograph by Rachel Armstrong, Milan Triennale, 2025.

This playground installation treats soil as a living archive, anticipating the ecosystems today's children will inherit (Figure 12). Soils transplanted from warmer regions like Puglia are considered ‘pre‐adapted’—meaning they have already adjusted to a hotter, drier climate that Milan is projected to experience by 2100. With an estimated +5°C temperature increase and climate zones shifting roughly 1 m per hour toward the poles, Puglia's microbial communities offer a natural preview of future conditions. As ecosystems adapt, so do microbial communities, affecting both environmental and human health. Embedding ‘future‐adapted’ soils into play spaces reframes public areas as probiotic zones, where microbial literacy begins in childhood through touch, breath and immersion. Soil becomes both a pedagogical tool and a living asset—an ecological primer for microbial futures.

##### 5.2.1.3 Lydia Kallipoliti and Hayley Eber—*Fatberg Autopsies*

**FIGURE 13:**
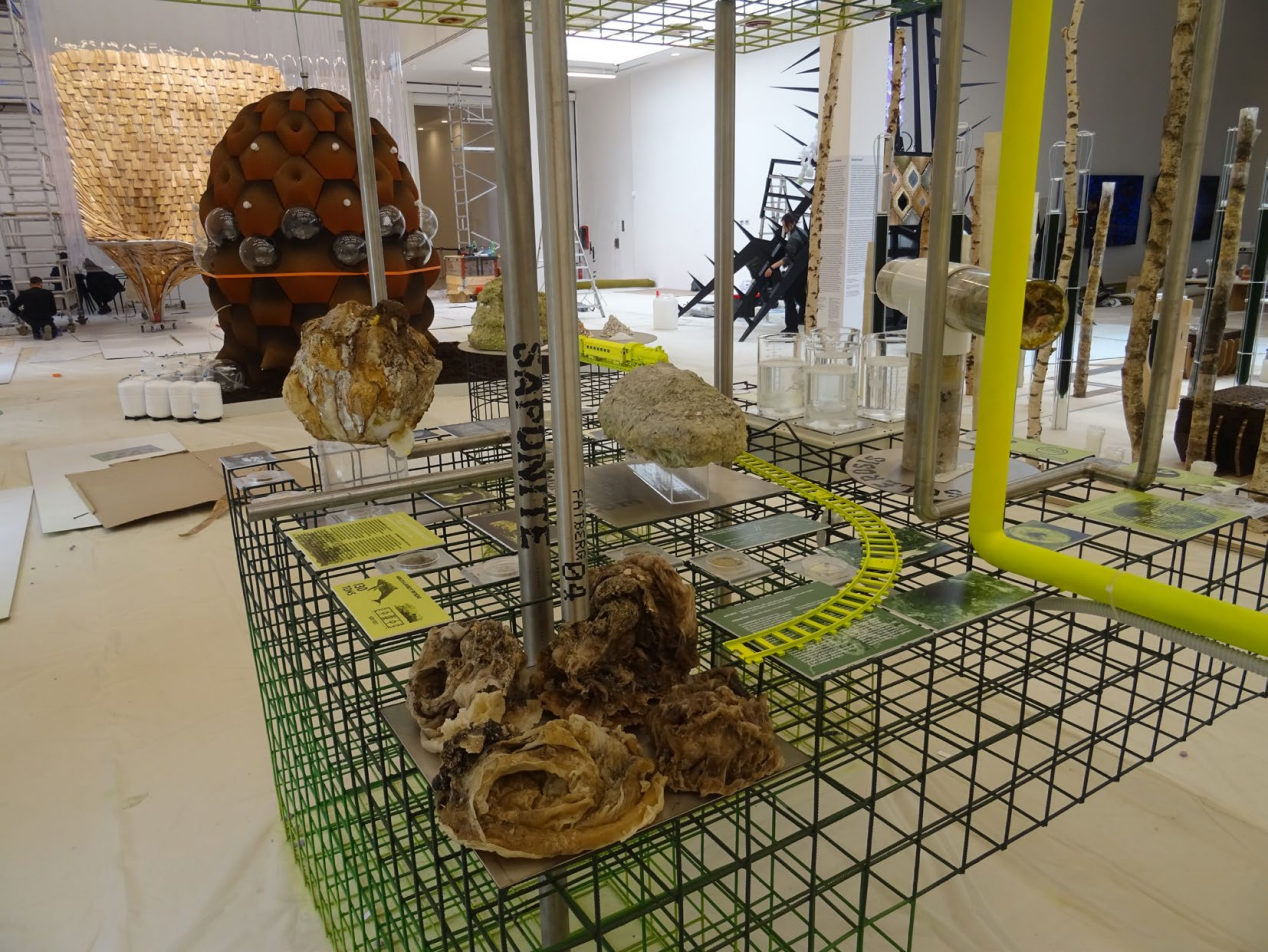
Fatberg Autopsies by Lydia Kallipoliti and Hayley Eber: Dissected urban fatbergs reveal waste‐metabolising bacteria, framing these waste formations as unintended laboratories for microbial resilience and bioremediation potential. Photograph by Rachel Armstrong, Milan Triennale, 2025.

Fatbergs—congealed masses of domestic waste—are dissected as microbial ecosystems, exposing the consequences of poor waste management and strained infrastructure (Figure 13). Yet, these urban accumulations also host bacteria that metabolise waste, offering insight into microbial resilience and potential for bioremediation. By linking fatbergs to soil, the installation highlights how domestic neglect disrupts microbial cycles, turning sewers into unintended Petri dishes of urban life.

#### 5.2.2 Architecture as a Microbial Partner

This group of projects rejects sterile modernism; instead, it designs buildings that collaborate with microbial life—making microbial labour visible, functional and central to sustainable design.

##### 5.2.2.1 David Benjamin—*Living Bricks*

**FIGURE 14:**
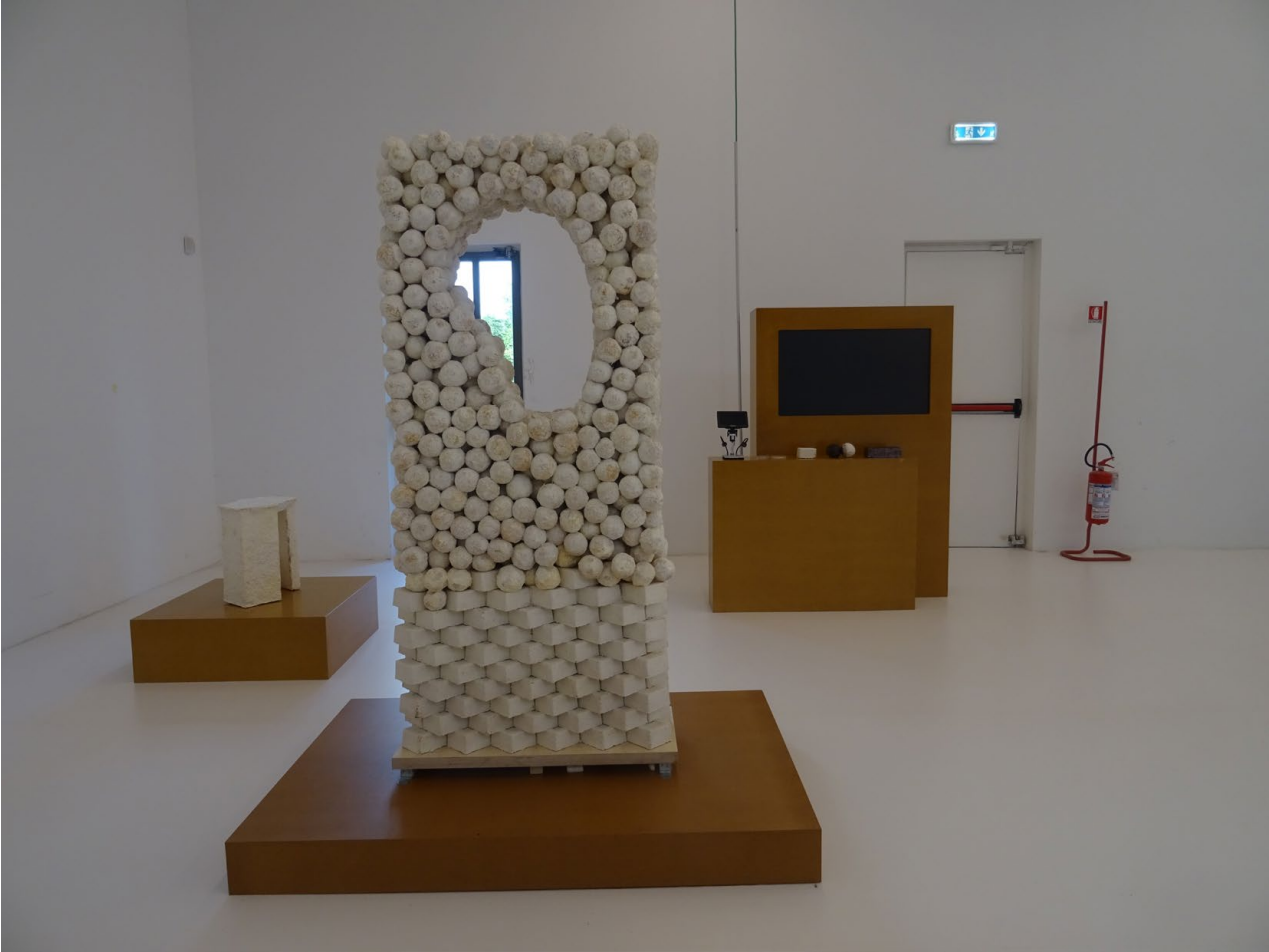
Living Bricks by David Benjamin: ‘Programmable’ microbial bricks and an adaptive chair demonstrate architecture as living systems, where bacteria and fungi enable self‐repair, environmental response and structural reconfiguration. Photograph by Rachel Armstrong, Milan Triennale, 2025.

David Benjamin's *Living Bricks* installation explores architecture as a living, adaptive system by integrating bacteria and fungi into programmable building components and dynamic furniture (Figure 14). The modular bricks are embedded with microbial systems that enable them to grow, self‐repair and adapt to environmental conditions—demonstrating how construction materials can become metabolically active. These living systems are not merely decorative but are designed to respond to contextual cues such as substrate composition, humidity or temperature, enabling functional reconfiguration over time. The adaptable chair similarly incorporates microbial collaboration, allowing it to change in form or firmness in response to use or environmental stimuli. Together, these components challenge the notion of buildings and furniture as static objects, proposing instead a future where architecture is dynamic, symbiotic and ecologically resilient. By embedding microbial labour into the fabric of design, Benjamin's work exemplifies a transition toward metabolic collaboration—where sustainability emerges through partnership with living systems rather than resource extraction.

##### 5.2.2.2 EcoLogic Studio—*DeepForest^3^*

**FIGURE 15:**
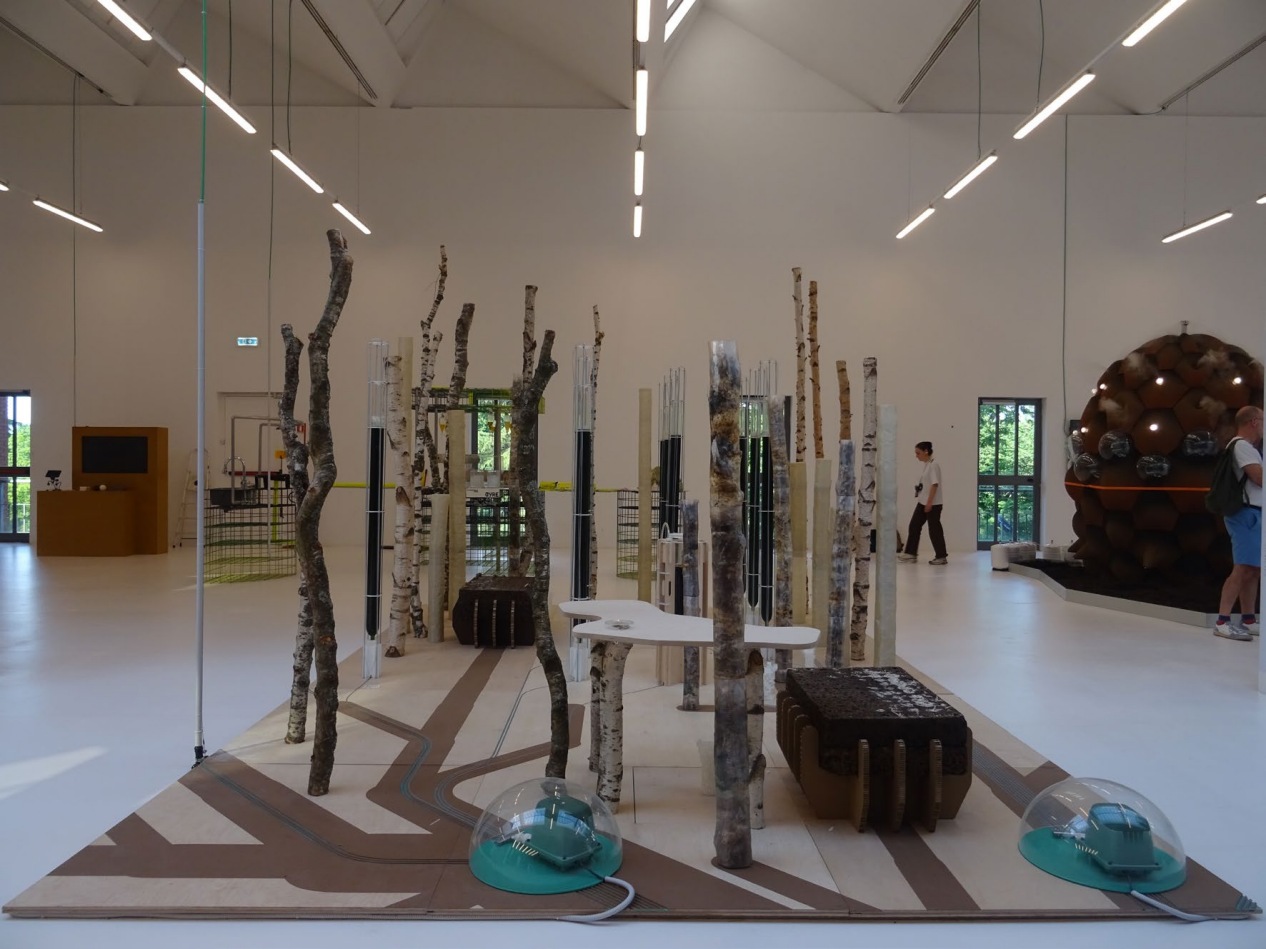
DeepForest^3^ by EcoLogic Studio: A living home integrating cyanobacteria and mycelium‐infused walls creates a symbiotic architecture that captures CO_2_, grows biodegradable structures and purifies air through microbial collaboration. Photograph by Rachel Armstrong, Milan Triennale, 2025.

*DeepForest* ^3^ combines domestic living with biological systems, integrating photosynthetic cyanobacteria and mycelium‐infused walls (Figure 15). Tree roots, algae‐based biopolymers and salvaged birch create a porous, living structure. Cyanobacteria capture CO_2_, while mycelium—grown on recycled coffee grounds—forms biodegradable architectural elements. The result is a symbiotic home that purifies air, regenerates materials and promotes microbial literacy through everyday cohabitation.

##### 5.2.2.3 Daniela Mitterberger & Tiziano Derme—Daphne's Skin

**FIGURE 16:**
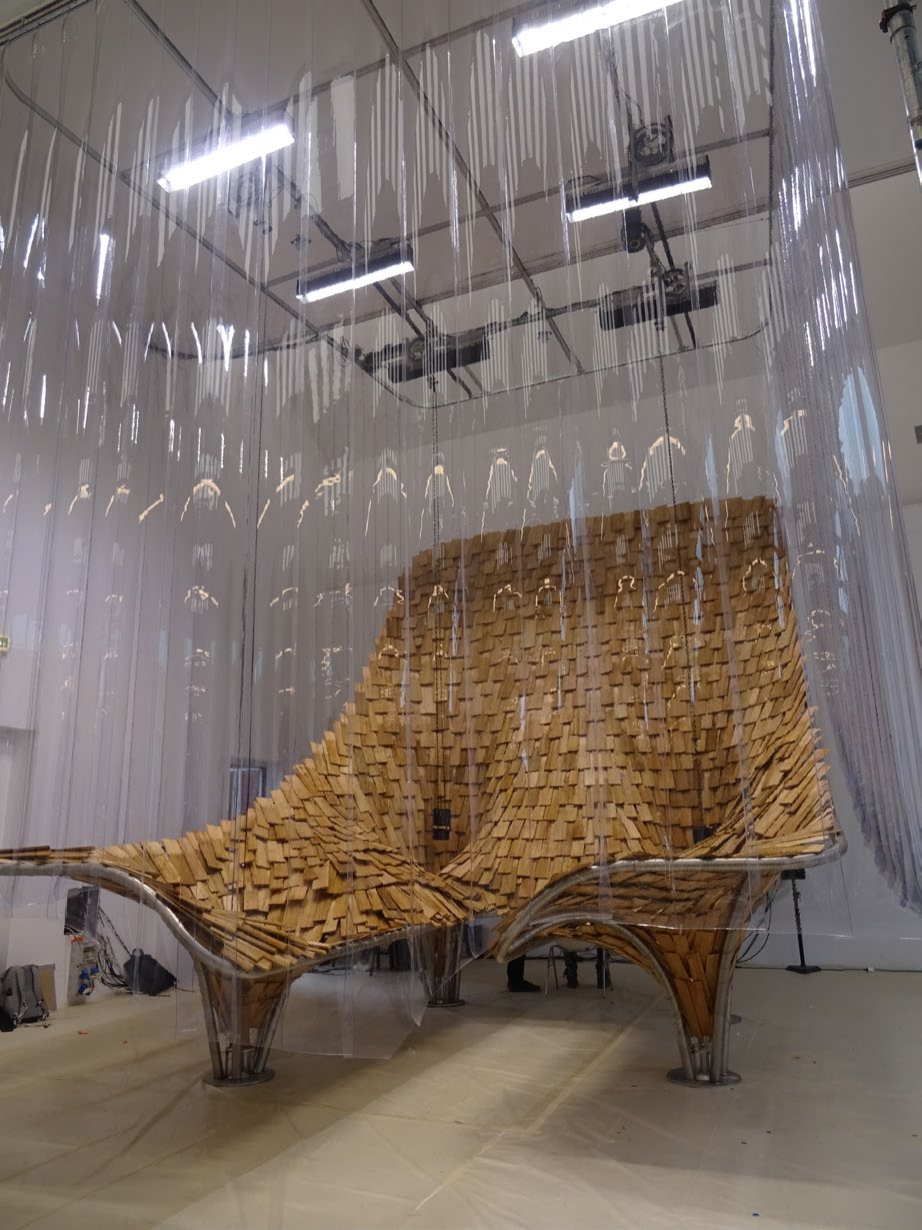
Daphne's Skin by Daniela Mitterberger & Tiziano Derme: A biologically active wooden surface colonised by desert algae and nitrogen‐fixing bacteria grows a living patina, with robotic systems modulating conditions to create an adaptive architectural skin where biology and technology co‐evolve. The culture is restrained behind a transparent curtain. Photograph by Rachel Armstrong, Milan Triennale, 2025.

Inspired by the myth of Daphne, this installation features a biologically active wooden surface seeded with *Tetradesmus deserticola* and *Azospirillum brasilense* (Figure 16). A green microbial patina spreads across the wood, monitored by robotic ‘geographers’ that adjust light and humidity. The result is a responsive architectural ‘skin’—where microbes, machines and materials co‐create a living interface.

##### 5.2.2.4 Rachel Armstrong—*SPIKA*

**FIGURE 17:**
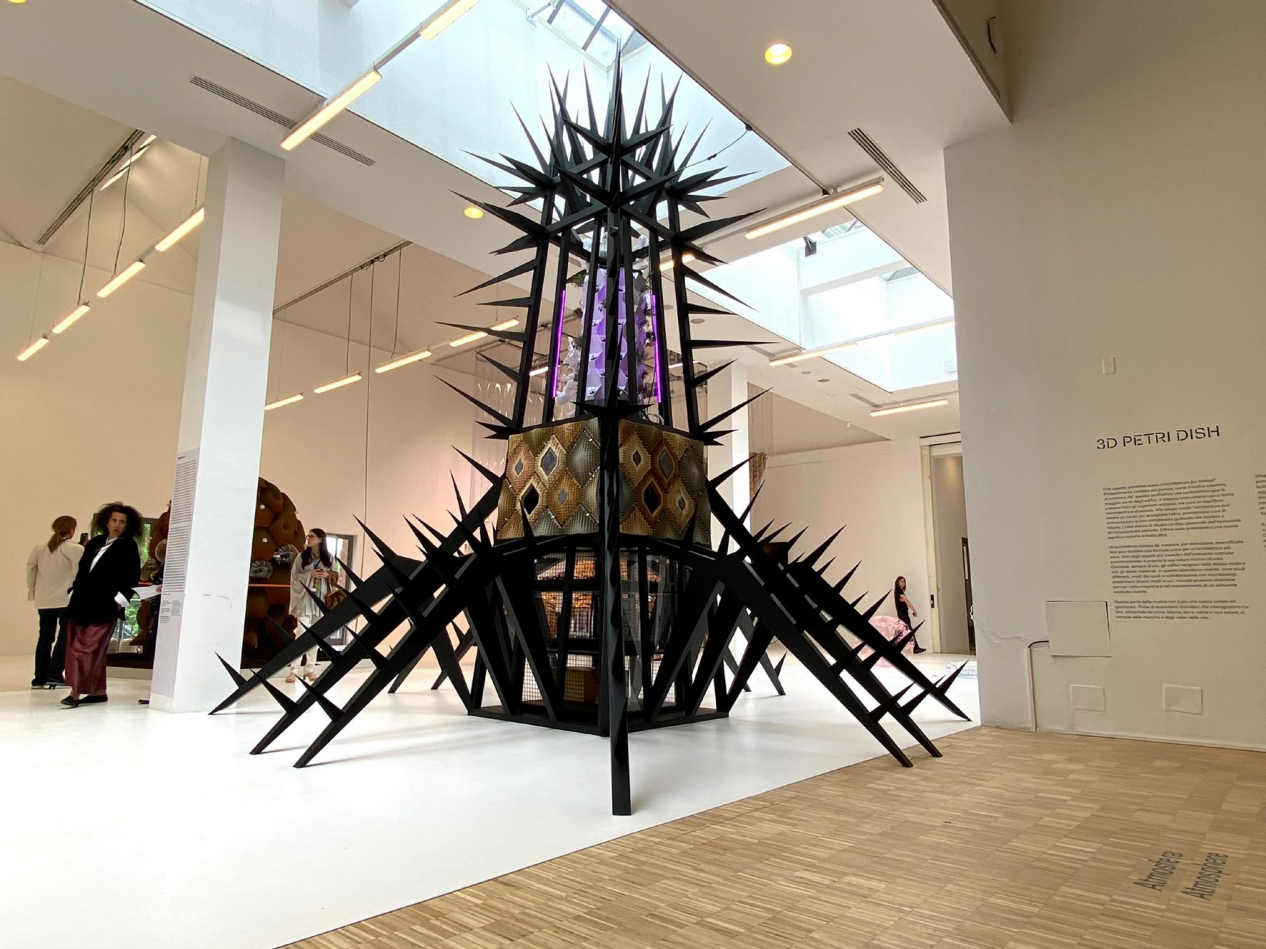
SPIKA by Rachel Armstrong: A bacteriophage‐inspired structure housing microbial fuel cells and hydroponics transforms waste into energy and nutrients through electrogenic biofilms and bioreceptive plant systems, reimagining architecture as a regenerative ecological partner. Photograph by Rachel Armstrong, Milan Triennale, 2025.

SPIKA (Structural Protection for Interdependent Karyotype Assembly) reimagines building systems and utilities services through microbial ecology and design (Figure 17). Inspired by bacteriophages, its thorn‐like form protects internal ecosystems while provoking reflection on human disruption. At its core is the *Microbial Hydroponics: Circular Sustainable Electrobiosynthesis* (Mi‐Hy) system, which integrates microbial fuel cells (MFCs) and hydroponics to convert household waste into energy, nutrients and materials (CO2NITROGEN, n.d.; Mi‐Hy, n.d.). Internally, electrogenic biofilms—powered by *Geobacter* species—generate electricity, while externally, bioreceptive surfaces support the growth of microalgae and mosses. These early colonisers represent the first stages of ecological succession, paving the way for more complex plant life. This time‐based pathway runs from the ground (microbes) to the panels (rootless plants) and on to the hydroponics systems with complex plants at the top of the structure.

SPIKA incorporates a prosthetic rhizosphere—a synthetic root environment that mimics the microbial interactions typically found in soil. In hydroponic systems, where plants lack access to native soils, MFCs play a crucial role by producing vital biomolecules that enhance plant health, flavour and nutritional value. This microbial support enables the transition from rootless to rooted plants, completing a tertiary level of succession within the architectural system.

By embedding microbial and plant life into the structure itself, SPIKA transforms architecture into a living, metabolic core—capable of processing waste, closing resource loops and reducing reliance on fossil fuels. It reimagines buildings as dynamic agents in ecological cycles, capable of producing energy, purifying water and sustaining life through microbial‐plant collaboration.

#### 5.2.3 Multi‐Species Cohabitation

These works explore microbial‐human entanglements as social practice, challenging human exceptionalism and promoting solidarity across species and geographies.

##### 5.2.3.1 Andrés Jaque—*Transspecies Palace*

**FIGURE 18:**
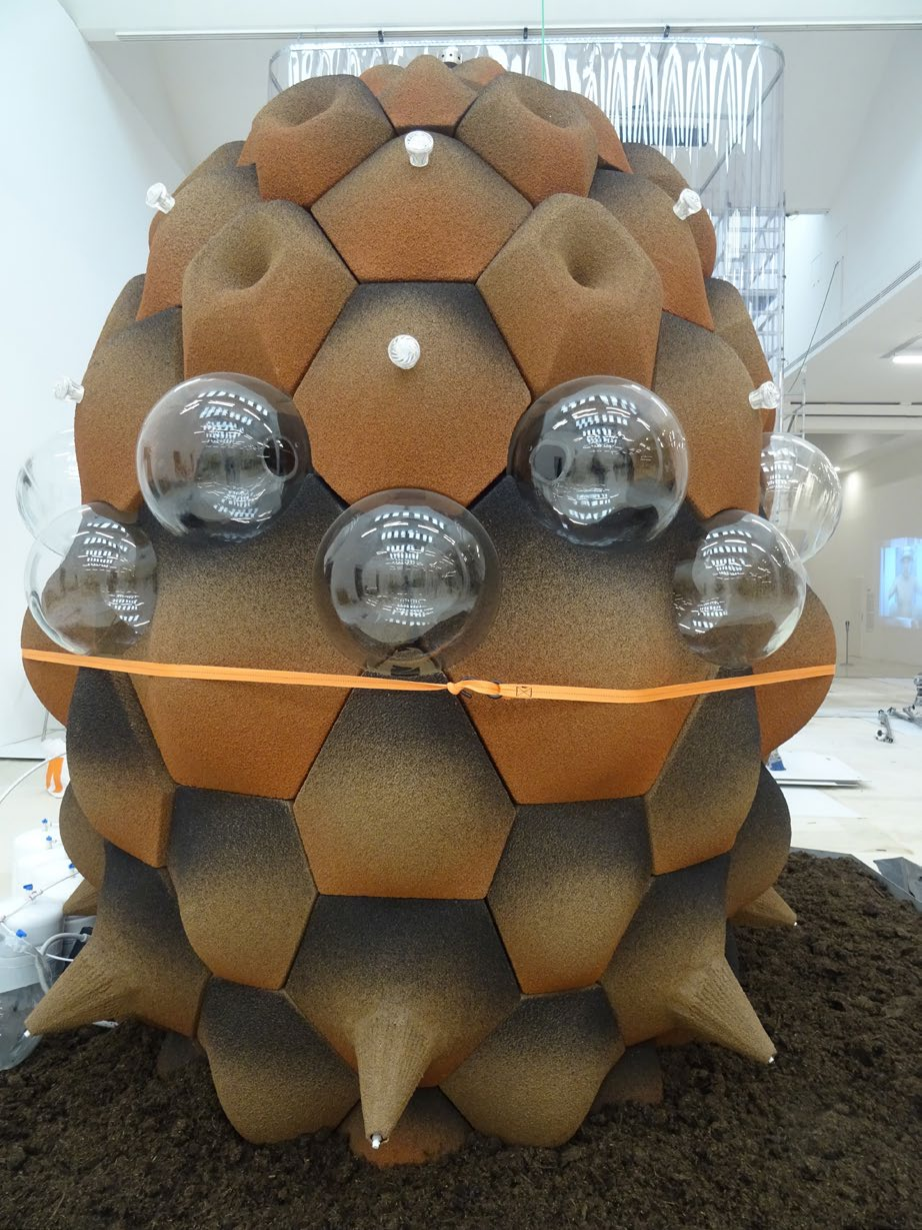
Transspecies Palace by Andrés Jaque: A cork bioreactor wall cultivates cyanobacteria, nitrogen‐fixing microbes and fungal networks to transform architecture into a shared respiratory system, where microbial carbon capture and human breathing co‐evolve within a living material matrix. Photograph by Rachel Armstrong, Milan Triennale, 2025.

This cork‐walled bioreactor reimagines breathing as a shared planetary act (Figure 18). Originally developed for the Reggio School in Madrid, it serves as a living interface where microbial, material and human systems co‐evolve. Known as the *Transspecies Palace*, the installation hosts a diverse microbial community—including cyanobacteria, Proteobacteria, Firmicutes and nitrogen‐fixing species such as *Azotobacter* and *Rhizobium*—embedded within a porous cork matrix. Dead fungal filaments provide structural anchorage for bacterial growth and exopolysaccharide formation, transforming the wall into a living scaffold for carbon capture and oxygen release. In this way, respiration becomes a distributed process—shared across surfaces—linking microbial metabolism with human breath in a continuous ecological exchange.

##### 5.2.3.2 Orkan Telhan & Elii—*Salivations*

**FIGURE 19:**
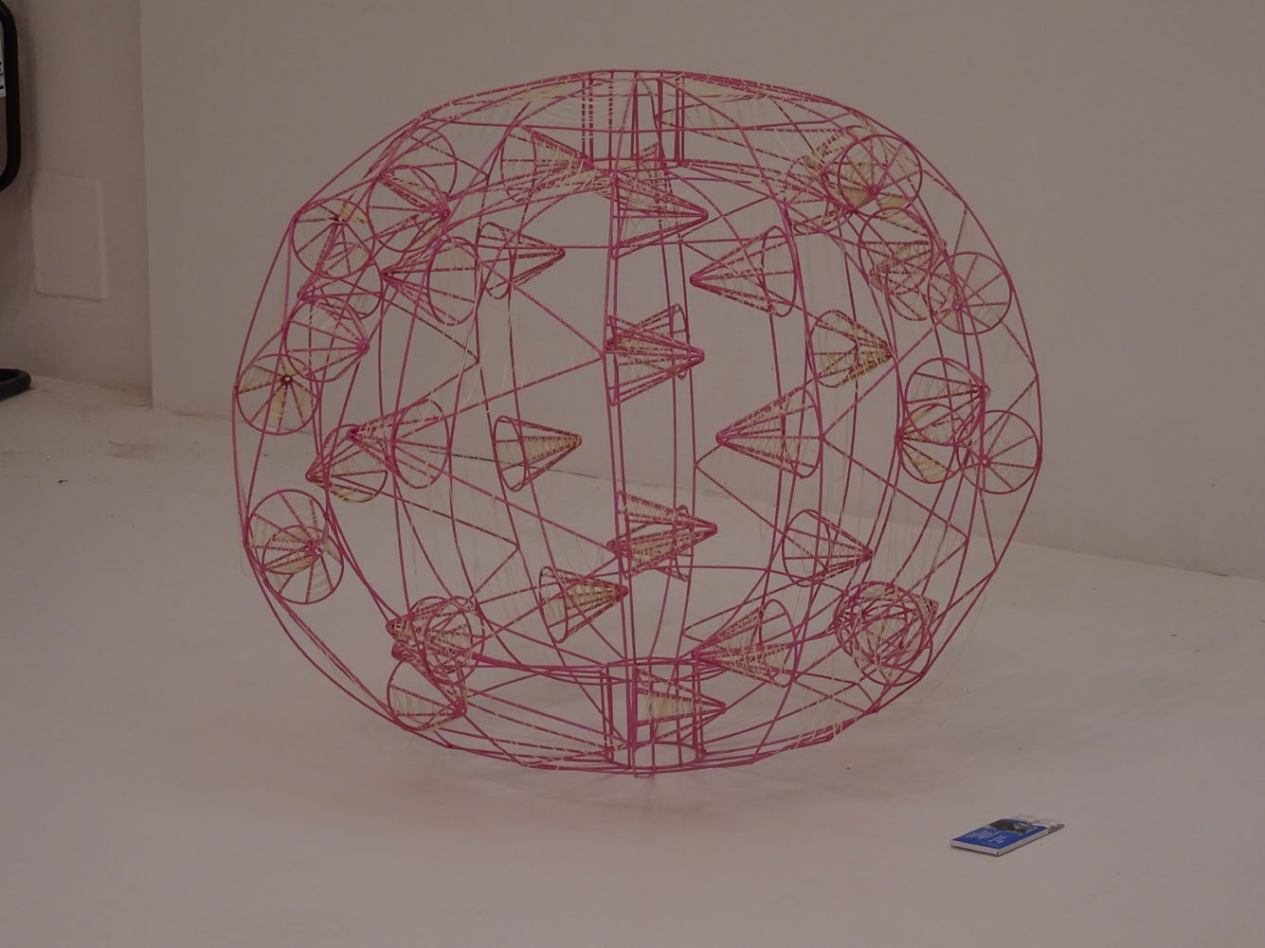
Salivations by Orkan Telhan & Elii: Wire‐frame chew toys and gum sculptures map human‐canine microbial exchanges via saliva, revealing how shared environments and migration histories shape overlapping oral microbiomes—transforming spit into a transspecies archive of care, displacement and co‐evolution. Photograph by Rachel Armstrong, Milan Triennale, 2025.

*Salivations* explores microbial intimacy between humans and dogs through saliva as a fluid archive (Figure 19). Using wire‐frame models of dog chew toys and chewing gum from canine companions—Çaça, Bailey, Kahve and Gatsby—the installation traces microbial exchanges shaped by migration, adoption and shared environments. DNA sequencing reveals overlapping oral microbiomes, shaped not only by proximity but by histories of movement, domestication and multispecies cohabitation. Saliva functions as a transspecies medium, carrying microbial memory and political histories—from Turkey's displacement of stray dogs to U.S. pet ethics. These chew toys, saturated with saliva and scent, become vessels of microbial transmission and embodied memory. Rather than symbolic gestures of care, they materialise the ethical entanglements of living together—where empathy arises not from sentiment but from an awareness of shared biological and environmental interdependence. By foregrounding microbial exchange as a site of relational ethics, *Salivations* invites reflection on how multispecies intimacy—however mundane—can reveal the invisible infrastructures that bind us to our nonhuman companions, regardless of origin, status or species.

#### 5.2.4 Interruption: On Cleaning (An Allegory)

**FIGURE 20:**
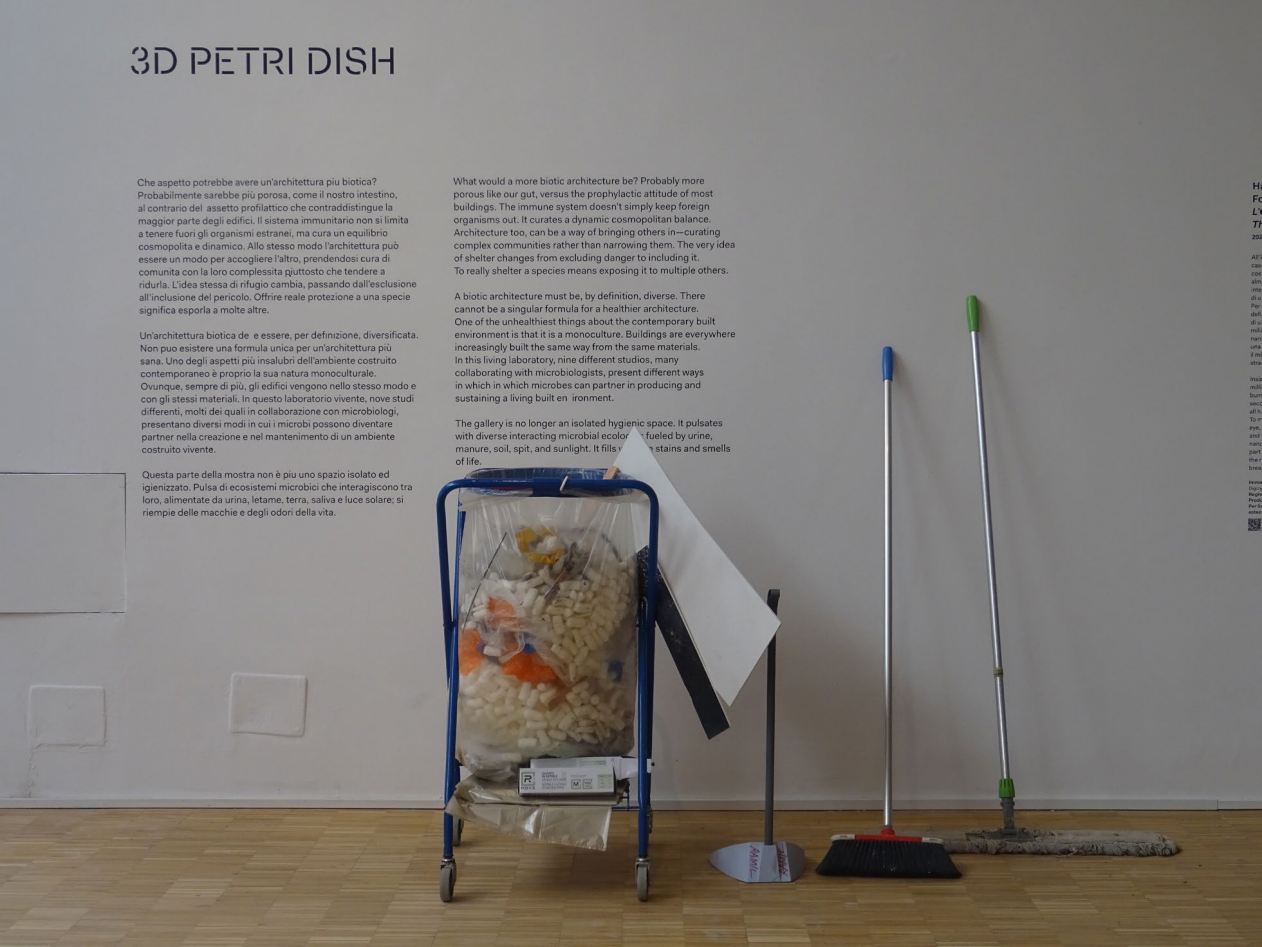
While designers build with microbes, cleaners decide if they survive. Cleaning materials—tools for navigating the 3D Petri Dish laboratory/gallery space, the gateway to hygiene, photograph by Rachel Armstrong, Milan Triennale, 2025.

A chance observation of a cleaner moving through the *We the Bacteria* exhibition revealed a quiet contradiction—one that serves as an allegory for institutional tensions around microbial life. As surfaces were wiped and bins emptied, hygiene rituals exposed the paradox of celebrating microbial agency while simultaneously fearing contamination. The mop and disinfectant became silent symbols of this contradiction, highlighting the challenge of promoting microbial literacy within sanitised institutions (Figure 20). Puglia's climate‐shifted soils and Andrés Jaque's *Transspecies Palace* invite microbial coexistence, yet nightly cleaning routines may erase the very microbiomes these works encourage. This mirrors a broader cultural double standard: we admire microbial intelligence in EcoLogic Studio's *DeepForest* ^*3*^ or MAEID's patinas, yet scrub mould from showers. As allegory, the cleaner's actions do not assign responsibility but instead reflect the institutional ambivalence toward microbial life. While decision‐makers shape policies around microbial coexistence, frontline staff—like cleaners—become inadvertent mediators of these norms. A truly probiotic approach would equip all stakeholders—from architects to custodians—with the tools to distinguish harmful pathogens from beneficial colonies, aligning practice with principle.

Rather than viewing cleaning as antagonistic, a truly probiotic approach would empower maintenance staff as microbial stewards—able to distinguish between harmful pathogens and beneficial colonies. The mop becomes a tool of microbial diplomacy, the cleaner a mediator of multispecies futures. The real test of microbial literacy lies not in spectacle, but in whether its principles shape the routines of those who hold the mops and buckets.

## 6 From Showcase to Shared Knowledge: The Role of Exhibitions in Expanding Microbial Literacy

High‐profile exhibitions such as *We the Bacteria: Notes Toward a Biotic Architecture* exemplify the creative and cultural potential of microbial storytelling. While these installations are resource‐intensive, they are not presented as the exclusive or ideal mode of public engagement. Rather, they function as experimental platforms—pushing the boundaries of material practice, sensory experience and curatorial framing. By embedding microbial narratives within broader societal themes, such as inequality—as reflected in the Milan Triennale's overarching focus—these exhibitions highlight the relevance of microbes to both human and planetary health.

Importantly, the *We the Bacteria* installations do more than raise awareness; they prototype new ways of living with microbes that align with several of the United Nations *Sustainable Development Goals* (Table 1). As evidenced in the table, each installation contributes to multiple SDGs simultaneously, underscoring the systemic relevance of microbial relationships across domains such as health, climate, food and education. This highlights the foundational role of microbial literacy—not merely as a scientific competency, but as a prerequisite for developing integrated, sustainable solutions. Cultivating this literacy is essential for enabling the multisectoral transformations envisioned by the SDGs. For instance, by illustrating the role of the microbiome in regulating immunity, metabolism and mental health, the exhibition contributes to SDG 3, which promotes good health and well‐being. The integration of microbial systems into architectural materials and urban design speaks directly to SDG 11, which calls for sustainable cities and communities. In showcasing microbial innovations such as bioplastics and circular material systems, the exhibition supports SDG 12, focused on responsible consumption and production. The emphasis on microbial contributions to soil regeneration, carbon cycling and climate‐responsive design aligns with SDG 13, which addresses climate action. Finally, the exhibition's potential to be adapted into community workshops, open‐access resources and digital learning tools reflects a commitment to SDG 4, which promotes inclusive and equitable quality education.

**TABLE 1:** *We the Bacteria* installations and their SDG alignment.

Project/Artist	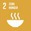	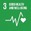	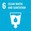	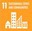	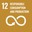	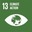	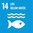	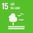	Key themes
SDG	2	3	6	11	12	13	14	15	SDG
Terra Preta: Paolo Tavares & Studio Autonoma	✓	✓		✓		✓	✓	✓	Microbial ecology, climate resilience
Living Bricks: David Benjamin		✓		✓	✓	✓		✓	Biofabrication, circular materials
Climate‐Shifted Playgrounds: Philippe Rahm	✓	✓		✓		✓		✓	Soil health, climate adaptation
Fatberg Autopsy: Lydia Kallipoliti & Hayley Eber		✓	✓	✓	✓			✓	Waste, pollution, hygiene
DeepForest^3^: Ecologic Studio		✓		✓		✓	✓	✓	Carbon sequestration, renewable energy, circular materials
Multispecies Palace: Andrés Jaque		✓		✓	✓		✓	✓	Multispecies architecture, biodiversity
Salivations: Orkan Telhan & Elii	✓	✓	✓	✓				✓	migrant histories through dog/human saliva microbiomes, kinship in microbial exchange
Daphne's Skin: Daniela Mitterberger & Tiziano Derme (MAEID)	✓	✓		✓	✓			✓	Bio‐digital interfaces, sustainable building materials
SPIKA: Rachel Armstrong & Microbial Hydroponics		✓	✓	✓	✓	✓		✓	Closed‐loop resource systems, tasty food production, microbial biofactory platform, C/N management and valorisation

Assessing the impact of exhibitions on microbiology literacy requires a combination of qualitative and quantitative methods that capture both cognitive and affective dimensions of learning. Given the interdisciplinary and experiential nature of exhibitions like *We the Bacteria*, evaluation strategies must be equally multifaceted. Qualitative methods such as visitor interviews, focus groups and open‐ended surveys can offer valuable insights into how participants interpret microbial concepts, reflect on their entanglement with microbial life and engage with the ethical, ecological and aesthetic dimensions of the installations. In *Salivations*, for example, interviews or interactive questionnaires could explore how visitors understand microbial intimacy and multispecies ethics through the medium of shared saliva and oral microbiomes. This approach resonates with *Gut Feelings*, where personal microbiome data was used to make invisible microbial connections tangible—bridging the gap between internal microbial ecosystems and individual identity through data‐driven storytelling. Similarly, in SPIKA, qualitative feedback could assess whether visitors grasp the role of microbial fuel cells and prosthetic rhizospheres in regenerative design for buildings. Observational studies offer another layer of insight. By tracking how visitors move through the space, interact with installations and engage with interpretive materials, curators can assess which elements promote curiosity, confusion, or sustained attention. For instance, observing how long visitors spend at *DeepForest* ^*3*^'s living spaces can reveal the effectiveness of spatial storytelling. On the quantitative side, engagement analytics—such as dwell time, interaction frequency and digital participation (e.g., QR code scans, AR features or social media shares)—can provide measurable indicators of impact. These metrics can be correlated with specific learning goals, such as increased awareness of microbial roles in sustainability or improved understanding of microbiome diversity. Together, the implementation of these methods can form a targeted, robust framework for evaluating how exhibitions contribute to microbiology literacy. Importantly, they also support the goals of initiatives like IMiLI, which advocate for accessible, student‐centred learning materials that extend beyond the gallery into classrooms and communities.

While the primary focus of the exhibition space or gallery, remains on microbial literacy, the dissemination, innovation and prototyping embedded in these works offer scalable insights for addressing global challenges. As initiatives like *Gut Feelings* have shown, galleries alone cannot reach all audiences. Their geographic and economic limitations require a distributed approach to engagement. The knowledge generated within exhibitions—through images, stories and design strategies—can be extended through open‐access publications, community workshops, mobile and digital platforms, as envisioned by IMiLI. In this way, exhibitions are not endpoints but catalysts. They initiate conversations, alter perceptions and provide frameworks that others—scientists, artists, educators and activists—can adapt and expand. Whether through a prestigious biennale or a local pop‐up laboratory, the goal remains the same: to make microbial literacy accessible, actionable and relevant. By combining high visibility showcases with grassroots dissemination, these exhibitions contribute meaningfully to a broader cultural transformation—one that embraces microbial life as central to sustainable futures.

## 7 Summary: Exhibition‐Based Microbial Pedagogy

How should we learn about something as invisible, complex and ancient as microbial life? The case studies in this opinion piece suggest that understanding microbes requires more than facts—it demands sensing, engaging and rethinking our relationship with them. Learning here is not linear or didactic—it is spatial, sensory and situated. From Simonides to Gibson, spatial learning theories remind us that knowledge is staged, not simply transmitted. Microbial exhibitions function as contemporary ‘theatres of activity,’ where visitors encounter microbes through designed affordances rather than abstract explanation. This aligns with IMiLI's vision of societally relevant education, where empirical and experiential knowledge converge to make the microbial realm legible.

Exhibitions like *We the Bacteria* and *Gut Feelings* show that microbial literacy is not just about knowing microbes exist—it is about sensing their presence, understanding their agency and recognising our entanglement with them across space, time and matter. Galleries collapse microbial deep time into moments of visceral recognition, becoming sites of ecological empathy and evolutionary awareness.

The exhibition format offers a unique pedagogical advantage: learning through interfaces. These are spaces where microbial processes become tangible, affective and participatory. With organisms too small to see and histories too vast to grasp, learning becomes a matter of *cultivating attunement*—an embodied sensitivity to microbial systems and scales. In *Exploring the Invisible*, bioluminescent light reveals microbial presence through atmosphere. In *Transspecies Palace* and *Salivations*, breath and saliva become media of microbial exchange, blurring the boundaries between self and environment. These works choreograph encounters with microbes.

Exhibitions also engage microbial deep time. In *∞ Blue*, designer scents and cyanobacterial structures evoke primordial landscapes, while *Fatberg Autopsies* reveal microbial accumulations from urban waste—slow, sticky archives of domestic excess. *Terra Preta* draws on millennia of Indigenous soil stewardship, while climate‐shifted playgrounds and SPIKA show microbial life as inseparable from air, humidity, temperature and architecture. *Deep Forest* ^*3*^ and David Benjamin's microbial bricks integrate microbes into everyday materials, teaching that to learn about microbes is to learn about the conditions that support life.

Yet exhibitions also reveal the limits of their pedagogy. The cleaner—moving through the gallery with broom and disinfectant—serves as an allegorical figure, embodying the tension between microbial celebration and institutional sanitisation. Their presence raises symbolic questions about access: who gets to learn, and who is tasked with erasing microbial traces? This allegory does not assign responsibility to the cleaner but instead highlights the structural contradictions within cultural institutions. If microbial literacy is to extend beyond the gallery, it must include those who maintain our spaces—not just those who curate them. A truly inclusive approach would recognise the roles of all participants in shaping multispecies futures, from educators to custodians.

To deepen their impact, exhibitions must embrace interdisciplinary, experiential learning. Scientists, artists, designers and curators should collaborate to convey the social, ethical and aesthetic dimensions of microbiology—realms that transcend data but remain grounded in it. Exhibitions can contribute to microbial literacy by bridging science, culture and society—but they must also confront their own exclusions—of labour, knowledge systems and species. While exhibitions such as the Milan Triennale may be limited by geographic location or entry fees, the ideas and installations they showcase often transcend these boundaries. Many of the works are widely documented and disseminated, offering valuable resources for classrooms, community spaces and informal learning environments. Initiatives like IMiLI support this broader access by promoting open, student‐centred materials that integrate science with culture and design. In this way, exhibitions can serve as catalysts for microbial literacy beyond the gallery—extending their reach into everyday educational practices and public imagination.

Ultimately, the microbial literacy embodied in these exhibitions is not just a tool for making sustainable buildings or living healthier lives—it is a system for knowledge distribution through storytelling that enables a new way of being. The challenge is not only to see the microbial world, but to live differently because of it. This transition calls for more than scientific understanding; it demands cultural transformation. By learning to design with, live alongside, recount and negotiate our relationship with microbial life, we begin to reimagine our place within a deeply interconnected biosphere—a world of symbiosis, storytelling, value‐making and stewardship. Through microbial literacy, we are invited to cultivate not only new materials, hygiene rituals and production methods, but also to establish strong environmental ethics, life‐promoting aesthetics and probiotic ways of inhabiting the world.

## Author Contributions

**Rachel Armstrong:** conceptualization, writing – original draft, visualization, writing – review and editing.

## Disclosure

The author reports being a featured exhibitor in the *We The Bacteria: Notes Toward a Biotic Architecture* exhibition (2025).

## Conflicts of Interest

The author declares no conflicts of interest.

## Data Availability

The data that support the findings of this study are available online and are in the public domain.
